# Immune Abnormalities in Autism Spectrum Disorder—Could They Hold Promise for Causative Treatment?

**DOI:** 10.1007/s12035-017-0822-x

**Published:** 2018-01-06

**Authors:** Dominika Gładysz, Amanda Krzywdzińska, Kamil K. Hozyasz

**Affiliations:** Department of Pediatrics, Institute of Mother and Child, Warsaw, Poland

**Keywords:** ASD, Autism, Cytokine, Chemokine, Lymphocytes, Immune system

## Abstract

Autism spectrum disorders (ASD) are characterized by impairments in language and communication development, social behavior, and the occurrence of stereotypic patterns of behavior and interests. Despite substantial speculation about causes of ASD, its exact etiology remains unknown. Recent studies highlight a link between immune dysfunction and behavioral traits. Various immune anomalies, including humoral and cellular immunity along with abnormalities at the molecular level, have been reported. There is evidence of altered immune function both in cerebrospinal fluid and peripheral blood. Several studies hypothesize a role for neuroinflammation in ASD and are supported by brain tissue and cerebrospinal fluid analysis, as well as evidence of microglial activation. It has been shown that immune abnormalities occur in a substantial number of individuals with ASD. Identifying subgroups with immune system dysregulation and linking specific cellular immunophenotypes to different symptoms would be key to defining a group of patients with immune abnormalities as a major etiology underlying behavioral symptoms. These determinations would provide the opportunity to investigate causative treatments for a defined patient group that may specifically benefit from such an approach. This review summarizes recent insights into immune system dysfunction in individuals with ASD and discusses the potential implications for future therapies.

**Electronic supplementary material:**

The online version of this article (10.1007/s12035-017-0822-x) contains supplementary material, which is available to authorized users.

“The human body is a machine which winds its own springs”

Julien Offray de La Mettrie (eighteenth-century French physician and philosopher)

Autism spectrum disorders (ASD), according to the International Statistical Classification of Diseases and Related Health Problems and the 5th edition of the Diagnostic and Statistical Manual of Mental Disorders [1, 2] criteria, belong to pervasive developmental disorders (PDD) and are characterized by the coexistence of primary symptoms across several areas: improper or impaired language and communication development, difficulties in social attachment and interactions, and occurrence of rigid, stereotypic and repetitive patterns of behavior and interests. ASD children require comprehensive care and the support of specialists from various fields [2–5]. Incidence rates of ASD are controverted and dependent on diagnostic criteria. The Centers for Disease Control and Prevention calculated that the overall prevalence of ASD in children aged 8 years in the USA equaled 1 out of 68 children [6]. It is a serious social problem and an increasing global burden with implications for public health services [7].

Numerous attempts to determine the etiology of ASD have been conducted; nonetheless, it remains largely elusive. It is considered that genetic, neurological, immunological, and environmental factors play a role in the development of ASD. Growing attention is being paid to neuroimmunology as dysregulation of immune responses may lead to impairments in neurodevelopment and numerous findings of altered immune system function in ASD individuals have been reported [8].

## Immunological background of ASD

The first suggestion of a link between the immune system and ASD was formed by Stubbs in 1976 because of undetectable rubella antibody titers after a rubella vaccine challenge in autistic children [9]. Several studies in animal models confirmed that an immune challenge during pregnancy results in behavioral abnormalities. Maternal immune activation was shown to activate a macrophage inflammatory state with increased M1 polarization [10], lead to up-regulation of interferon-gamma (IFN-γ) and interleukin (IL) 17a secreted by CD4^+^ T cells [11], and cause a systemic deficit of T regulatory cells (Tregs) [12].

Mice injected with valproic acid during their gestational period were found to have reduced social interactions and therefore are proposed as a mouse model of ASD. They have chronic glial activation and present with an inflammatory response as evidenced by increasing numbers of microglia and production of higher levels of proinflammatory cytokines when stimulated with lipopolysaccharides (LPS) [13].

Presence of maternal anti-fetal brain autoantibodies has been reported to play a role in ASD as well [14–20]. Monkeys exposed prenatally to human immunoglobulin G (IgG) derived from mothers of ASD children were found to exhibit stereotypies, hyperactivity [21], or impaired social behavior [22]. Similar results were observed in mice [23]. BTBR mice (as a mouse model of ASD) were found to have higher levels of serum IgG, immunoglobulin E (IgE), and anti-brain antibodies along with up-regulation of several cytokines [24].

Family history of autoimmunity has been reported as a risk factor for ASD in multiple studies [25–39]. A meta-analysis on this topic identified hypothyroidism, type 1 diabetes, rheumatoid arthritis, and psoriasis as a major family history burden [29, 38]. Maternal autoimmune diseases beginning during pregnancy can strongly impact risk of ASD in offspring as well [39].

An association between some alleles of human leukocyte antigens (HLA) and autoimmune diseases has been established. Several studies have revealed links between HLA and ASD, where autistic children were found to have a higher frequency of HLA-DRB1*11 allele and lower frequency of HLA-DRB1*03 allele [40]. Several other investigators reported on an association between HLA and ASD in different populations including Caucasian [41–45], Thai [46], Saudi Arabian [47], and Chinese [48]. An interesting association of HLA-G polymorphism with ASD, likely a consequence of prenatal immune activation, was reported by Guerini et al. [49]. HLA-DR4 in mothers was also reported as the ASD risk factor for their offspring [50]. Consideration of genetic polymorphisms in the HLA region is advised when studying immunopathology of the disease [51].

A presence of brain autoantibodies in children with ASD also suggests immunological involvement [52–63]. Severity of ASD, measured with Childhood Autism Rating Scale (CARS), was found to be correlated with serum anti-neuronal [54] and anti-ganglioside M1 antibodies [63]. Anti-brain antibodies have been found to correlate with more impaired cognitive functions, motor stereotypies [52, 57], irritability, and lower expressive language skills [53, 57].

An accumulation of evidence in favor of an immune pathomechanism has led to studies of neonatal and mid-gestational cytokines as early markers of ASD (Table 1). In a population-based case–control study, monocyte chemotactic protein-1 (MCP-1) was elevated and the chemokine RANTES (for regulated upon activation, normal T-cell expressed and secreted) was decreased in newborn peripheral blood retrieved from archives that collected dried bloodspots for screening purposes (obtained 24–48 h after birth). RANTES was also found to be down-regulated in children with developmental delays other than ASD along with macrophage inflammatory protein-1α (MIP-1α) [66]. Another approach to identify children at risk for ASD was proposed by Goines et al. [71], who showed that increased IFN-γ, IL-4, and IL-5 in pregnant women at 15 to 19 weeks of gestation was associated with increased risk of bearing a child with ASD. A study from the same center revealed that mid-gestational concentration of cytokines and chemokines (especially granulocyte macrophage colony-stimulating factor—GM-CSF, IFN-γ, IL-1α, and IL-6) was highest in mothers of ASD children with intellectual disability [64]. A high concentration of IL-4 was found to be associated with increased risk of severe ASD, while IL-1β correlated with mild to moderate ASD [65]. A series of studies by Abdallah et al. [67–70] demonstrated a strong association of multiple cytokines detected in material from newborn screening, as well as several chemokines (MCP-1, MIP-1α, RANTES) and growth factors (brain-derived neurotrophic factor—BDNF, neurotrophin—NT, transforming growth factor-beta—TGF-β), in both neonatal dried bloodspots and amniotic fluid [70]. Down-regulation of NT in dried bloodspots of ASD children was confirmed by Nelson et al. [72]. The search for potential ASD biomarkers is well underway [73, 74]; for an excellent review, see Anderson [75].

**Table 1:** Summary of studies on neonatal and gestational ASD immune-specific biomarkers

Number	Study	Country	Time of sample collection	Study group maternal age (years)	Study group gestational age	Groups	Females (%)	Perinatal history	Psychological tools	Study material	Cytokines	Chemokines	Growth factors	Other analytes	Main results
1	Jones et al. 2017 [64]	USA, Mexico	2000–2003	M: 30.01, SD: 5.67	x	ASD (*n* = 415), DD (*n* = 188), HC (*n* = 428)	ASD (17.3), DD (43.6), HC (17.1)	Unknown, apart from plurality and parity	DSM-IV	Mid-gestational serum	IL-1RA, IL-1β, IL-2, IL-6, IL-9, IL-10, IL-12, IL-18, IFN-γ, TNF-α	CXCL1 (GRO-α), CXCL8 (IL-8)	GM-CSF	x	ASD+ID vs. DD: ↑GM-CSF, TNF-α, IL-1α, IL-1β, IL-6, IFN-γ, IL-10, IL-1Ra, MCP-1
2	Krakowiak et al. 2017 [65]	USA	2003–2005, mean 7.8 ± 1.4 years from collection to analysis	No data	39.3 ± 1.8, 31–45	ASD (*n* = 214), DD (*n* = 27), HC (*n* = 62)	ASD (12), DD (41), HC (19)	Unknown	DSM-V, ADI-R, ADOS, ABC, MSEL, SCQ, VABS	Dried bloodspots, routine newborn screening	IL-1β, IL-2, IL-4, IL-5, IL-6, IL-10, IL-12, IFN-γ, TNF-α	CCL2 (MCP-1), CCL3 (MIP-1α), CCL4 (MIP-1β), CCL5 (RANTES), CCL11 (eotaxin), CXCL8 (IL-8), CXCL10 (IP-10)	x	x	severe ASD vs. mild ASD: ↑IL-4; severe ASD vs. HC: ↑IL-4; mild ASD vs. HC: ↑IL-1β
3	Zerbo et al. 2014 [66]	USA, Mexico	2000–2001	Mdn: 31, IQR: 28–34	Preterm (*n* = 9), term (*n* = 75)	ASD (*n* = 84), DD (*n* = 49), HC (*n* = 159)	ASD (13.1), DD (40.8), HC (12.6)	Unknown	DSM-IV	Dried bloodspots, routine newborn screening	IL-1β, IL-2, IL-4, IL-5, IL-6, IL-10, IL-12p40, IFN-γ, TNF-α	CCL2 (MCP-1), CCL3 (MIP-1α), CCL4 (MIP-1β), CCL5 (RANTES), CCL11 (eotaxin), CXCL8 (IL-8), CXCL10 (IP-10)	GM-CSF	x	ASD vs. HC: ↑MCP-1 ↓RANTES; DD vs. HC: ↓MIP-1α, RANTES
4	Abdallah et al. 2013 [67]	Denmark	1982–2000	< 30 (*n* = 95), 30–35 (*n* = 101), > 35 (*n* = 163)	Preterm (*n* = 32), term (*n* = 324), postterm (*n* = 3)	ASD (*n* = 359), HC (*n* = 741)	ASD (18.9), HC (19.7)	Parity, Apgar score, birth weight, congenital malformations	ICD-8, ICD-10	Dried bloodspots, routine newborn screening.	x	x	BDNF, NT-4, TGF-β	x	ASD children—BDNF around 10th percentile and NT-4 less likely in upper percentiles, female ASD children—TGF-β around 10th percentile,
5	Abdallah et al. 2013 [68]	Denmark	1982–2000	< 30 (*n* = 95), 30–35 (*n* = 101), > 35 (*n* = 163)	Preterm (*n* = 32), term (*n* = 324), postterm (*n* = 3)	ASD (*n* = 359), HC (*n* = 741)	ASD (18.9), HC (19.7)	Parity, Apgar score, birth weight, congenital malformations	ICD-8, ICD-10	Dried bloodspots, routine newborn screening.	x	CCL2 (MCP-1), CCL3 (MIP-1α), CCL5 (RANTES)	x	x	Females with ASD: ↑RANTES
6	Abdallah et al. 2013 [68]	Denmark	1982–2000	< 30 (*n* = 109), 30–35 (*n* = 120), > 35 (*n* = 185)	Preterm (*n* = 43), term (*n* = 368), postterm (*n* = 3)	ASD (*n* = 331), HC (*n* = 698)	ASD (19.1), HC (19.5)	Parity, Apgar score, birth weight, congenital malformations	ICD-8, ICD-10	Amniotic fluid	x	CCL2 (MCP-1), CCL3 (MIP-1α), CCL5 (RANTES)	x	x	ASD vs. HC: ↑MCP-1
7	Abdallah et al. 2012 [69]	Denmark	1982–2000	< 30 (*n* = 109), 30–35 (*n* = 120), > 35 (*n* = 185)	Preterm (*n* = 43), term (*n* = 368), postterm (*n* = 3)	ASD (*n* = 331), HC (*n* = 698)	ASD (19.1), HC (19.5)	Parity, Apgar score, birth weight, congenital malformations	ICD-8, ICD-10	Amniotic fluid	x	x	BDNF, NT-4, TGF-β	MMP-9	ASD vs. HC: ↑MMP-9
8	Abdallah et al. 2012 [70]	Denmark	1982–2000	< 30 (*n* = 95), 30–35 (*n* = 101), > 35 (*n* = 163)	Preterm (*n* = 32), term (*n* = 324), postterm (*n* = 3)	ASD (*n* = 359), HC (*n* = 741)	ASD (18.9), HC (19.7)	Parity, Apgar score, birth weight, congenital malformations	ICD-8, ICD-10	Dried bloodspots, routine newborn screening.	IL-1β, IL-2, IL-4, IL-5, IL-6, sIL-6rα, IL-10, IL-12, IL-17, IL-18, IFN-γ, TNF-α, TNF-β	CXCL8 (IL-8)	GM-CSF	TREM-1	ASD vs. HC: ↓ IL-1β, IL-10; ASD children had GM-CSF, IFN-γ, IL-2, IL-4, IL-6 more likely around 10th percentile and IL-8 more likely around 90th percentile
9	Goines et al. 2011 [71]	USA, Mexico	x	M: 30.9, SD: 5.2	x	ASD (*n* = 84), DD (*n* = 49), HC (*n* = 159)	ASD (13.1), DD (40.8), HC (12.6)	Unknown, apart from plurality and parity	DSM-IV	Mid-gestational serum	IL-1β, IL-2, IL-4, IL-5, IL-6, IL-10, IL-12p40, IFN-γ, TNF-α	CCL2 (MCP-1), CCL3 (MIP-1α), CCL4 (MIP-1β), CCL5 (RANTES), CCL11 (eotaxin), CXCL8 (IL-8), CXCL10 (IP-10)	GM-CSF	x	ASD vs. HC: ↑IFN-γ, IL-4, IL-5; DD vs. HC: ↑IL-2, IL-4, IL-6
10	Nelson et al. 2006 [72]	USA	1998–1995	No data	No exact data	ASD (*n* = 47), DS (*n* = 46), HC (*n* = 90, including 28 preterm, 50 term, and 12 adults)	No data	Unknown	DSM-IV	Amniotic fluid	x	CXCL8 (IL-8)	BDNF	VIP, CGRP, NT-3, NT-4/5	ASD vs. HC: ↓NT-3

*M* mean, *SD* standard deviation, *Mdn* median, *IQR* interquartile range, *ASD* autism spectrum disorders, *DD* developmental delay, *HC* healthy controls, *DSM* Diagnostic and Statistical Manual of Mental Disorders, *ADI-R* Autism Diagnostic Interview–Revised, *ADOS* Autism Diagnostic Observation Schedule, *CARS* Childhood Autism Rating Scale, *MSEL* Mullen Scales of Early Learning, *SCQ* Social Communication Questionnaire, *VABS* Vineland Adaptive Behavior Scale, *ICD* International Statistical Classification of Diseases and Related Health Problems, *IL* interleukin, *IFN* interferon, *TNF* tumor necrosis factor, *TGF* transforming growth factor, *s* soluble, *R* receptor, *CXCL* C-X-C motif chemokine ligand, *GRO* growth-regulated oncogene, *MIP* macrophage inflammatory protein, *MCP* monocyte chemoattractant protein, *RANTES* regulated upon activation normal T-cell expressed and secreted, *CCL* C-C motif chemokine ligand, *IP-10* IFN-γ-inducible protein 10, *GM-CSF* granulocyte-macrophage colony-stimulating factor, *BDNF* brain-derived neurotrophic factor, *NT* neurotrophin, *MMP* matrix metalloproteinase, *TREM* triggering receptor expressed on myeloid cells, *VIP* vasoactive intestinal peptide, *CGRP* calcitonin gene-related peptide

ASD is very complex and heterogeneous. The question of whether immune dysregulation is a primary cause or secondary consequence is still open. Even if immune system integrity turns out to be a key player in ASD pathogenesis, it surely will not be the sole factor responsible for behavioral abnormalities. However, evidence for an immunological component is strong. It is worth noting that all published studies on neonatal and gestational immune mediators have succeeded in detecting some abnormalities in comparison to typically developing controls. However, attention should be given to some methodological concerns, such as lengthy times from sample collection to analysis, incomplete perinatal histories, lack of details on behavioral abnormalities, and cases of no clinical confirmation of ASD. Only the Krakowiak et al. study [65] confirmed ASD diagnoses and made an effort to evaluate behavioral traits. Interestingly, besides mediators classically associated with inflammation, an elevation of Th-2 cytokines was noted. An up-regulated concentration of serum mid-gestational IL-10 in mothers of autistic children was reported by Jones et al. [64]. These results should be interpreted with caution, however, as IL-10 is known to be physiologically elevated during pregnancy [76, 77] and its concentration was normal in another study from the same biological sample [71], and was not elevated [65, 66] or even down-regulated [70] in three other studies that utilized dried bloodspots from newborn screening. Surprisingly, another Th2 cytokine, IL-4, was found to be elevated in newborn dried bloodspots (acquired from children that were determined to have ASD) by Krakowiak et al. [65] who conducted the study with the best methodological quality. Moreover, high IL-4 level was connected with severe ASD. Another study found elevated IL-4 concentration in mid-gestational serum from mothers of children with ASD, which could be partially attributed to changes during pregnancy [76]. One of two experiments based on dried bloodspots did not detect any abnormalities [66], while another revealed tendency toward lower IL-4 concentrations in neonates that developed ASD [70]. Clearly, the purpose of these studies was to identify biomarkers that could precede occurrence of autistic traits. However, this task is very hard or nearly impossible due to several other perinatal factors that can alter results and due to methodological difficulties, particularly in confirming ASD diagnosis and finding connections between behavioral abnormalities and immune disturbances.

## Lymphocytes

One of the first clues concerning lymphocyte pathology in ASD was described by Stubbs and Crawford who found decreased lymphocyte response to stimulation with phytohemagglutinin (PHA) in children with ASD [78]. One of the first studies on lymphocyte subsets in ASD was carried out by Warren et al. in 1986 [79]. The investigators found a decreased number of T lymphocytes, reduced response to stimulation with PHA, concanavalin A, and pokeweed mitogen, and an imbalanced ratio of helper/suppressor cells. Another study confirmed lower helper/suppressor ratio with a decreased percentage of helper–inducer cells and decreased percentage of cells with expression of IL-2R after mitogenic stimulation being inversely correlated with severity of autistic traits [80].

Another early study on lymphocytes in ASD showed lower numbers of total lymphocytes in peripheral blood of ASD children compared to sibling and healthy controls, along with a significantly lower percentage and number of CD4^+^ helper T cells [81]. Ashwood et al. [82] reported significantly higher number of B cells in children with ASD aged 4–6 years in comparison with age- and sex-matched typically developing controls. The study protocol involved 64 three-color cellular assays that revealed higher counts of activated and mature B cells and higher numbers of cellular activation markers such as HLA-DR and CD26. The investigators further divided children into low and high functioning individuals according to intelligence quotient (IQ) measured with Stanford–Binet Intelligence Scale; however, there were no differences within subgroups. Ferrante et al. [83] observed a significant increase in CD4^+^ memory and decrease in CD4^+^ naïve T cells associated with HLA A2-DR11. Warren et al.’s studies [84, 85] on lymphocyte subpopulations revealed incomplete T cell activation, decreased numbers of lymphocytes and CD4^+^CD45RA^+^ cells, and normal levels of B, NK, and other T cells. Abnormalities regarding suppressor–inducer T cells have shifted researchers’ attention to T-cell biology in ASD.

Tregs play a key role in regulation of immune responses. A study on Egyptian children [86] revealed lower numbers of CD4^+^CD25^high^ Tregs in the blood of autistic children. Moreover, allergic problems and family history of autoimmunity turned out to be risk factors for the lowest number of CD4^+^CD25^high^ Tregs. The study was conducted on 30 patients and 30 age- and sex-matched healthy controls.

Dysregulation of Th1, Th2, Th17, and Treg-related transcription factors has also been described. Mononuclear cells derived from peripheral blood (PB-MNC) of autistic children and typically developing controls were stimulated and subsequently assessed for expression of mRNA and proteins of major transcription factors involved in neurodevelopment and differentiation of T cells. A deficit of forkhead box protein 3^+^ Tregs was found along with up-regulation of Th1/Th2/Th17-related transcription factors [87].

An imbalance of cytokines produced by CD4^+^ and CD8^+^ T cells with skewing toward Th2 response was found in 1997 by Gupta et al. [88]. Proportions of CD4^+^ and CD8^+^ T cells producing IFN-γ and IL-2 were reduced in opposition to T cells that produced IL-4. Further insight into Th2 response was provided by a study that concentrated on Th2 lymphocyte receptor ligands of 56 ASD children with 32 matched controls. Serum levels of macrophage-derived chemokine (MDC) and thymus and activation-regulated chemokine (TARC) were elevated and significantly correlated with intensification of autistic behaviors [89]. A higher percentage of CD8^+^ T cells (21.68% vs. 16.48%) and B cells (8.17% vs. 6.97%) and a decrease of CD4^+^/CD8^+^ ratio (3.01 vs. 3.97) was reported in 59 adult patients with ASD with a mean age of about 24 years versus a control group composed of 26 individuals, two of which were first-degree relatives, with no significant differences regarding age and gender of study participants [90].

Immune dysfunction is also observed in healthy siblings of ASD children, their immunophenotype is closer to their relatives than to typically developing matched controls. They have been found to have a higher concentration of cytokine-producing lymphocytes and CD8^+^ naïve T lymphocytes along with a down-regulation of CD8^+^ effector memory and CD4^+^ terminally differentiated lymphocytes [91].

Th17 CD4^+^ T cells are thought to be important players in autoimmune and neuroinflammatory diseases. Their product, IL-17A, is known to be up-regulated in several autoimmune diseases such as multiple sclerosis, systemic lupus erythematosus, and rheumatoid arthritis [92]. A cross-sectional study by Al-Ayadhi and Mostafa [93] on 45 children with ASD aged 6–11 years revealed a positive correlation of IL-17A with ASD severity. Nearly 50% of autistic children had elevated serum IL-17A levels, including 67.9% of children with severe and 17% of children with mild to moderate ASD. Up-regulation of IL-17 was also found in ASD children with concomitant asthma after T-cell stimulation with PHA [94]. A strong association of ASD with Th17 cells has also been demonstrated in animal studies, where effector cytokine IL-17a was essential for maternal immune activation and subsequent behavioral abnormalities [95]. IL-23, known to increase Th17 cell production of IL-17, was found to be down-regulated, especially in children with new-onset ASD, with no concomitant differences in IL-17 levels. Stimulation with PHA altered secretion of IL-23, which was found to be significantly lower than in typically developing controls and associated with more impaired behavioral scores [96, 97].

A BTBR mouse model of ASD was found to have up-regulated IgG production with IgG brain deposits and anti-brain IgG antibodies [98]. Several studies in humans have assessed concentrations of serum immunoglobulins and their subclasses in ASD individuals with discrepant results. A study on 15 subjects with ASD, 5 of whom underwent cerebrospinal fluid (CSF) immunoglobulin analysis, found no abnormalities [99]. Serum immunoglobulin A (IgA) deficiency was found by Warren et al. [100] in 40 individuals with ASD, both children and adults. Eight of 40 studied ASD patients had IgA levels below normal range adjusted for age, while in control groups there were no abnormalities. Mean serum IgA level of autistic individuals was significantly lower than in the control group (105 mg/100 mL vs. 143 mg/100 mL). Similar observations were made by Wasilewska et al. [101] who reported on lower IgA levels and up-regulated expression of CD23 on B lymphocytes derived from children with regressive ASD aged 3–6 years old. The studied groups consisted of 24 age- and gender-matched individuals with no differences regarding demographics and perinatal history.

In a study on 31 patients with selective IgA deficiency, 1 had a diagnosis of ASD [102]. The researchers focused on the offspring and siblings of the abovementioned group. Out of 87 children born to individuals with IgA deficiency, 3 had a diagnosis of ASD in comparison to 1 child out of 193 children born to subjects with normal IgA concentration. ASD was diagnosed in 2% of siblings (2/99 individuals) of IgA-deficient patients in contrast with 0.5% of siblings (1/217 individuals) in the control group. However, the abovementioned results did not reach statistical significance [102].

Analysis of plasma levels of immunoglobulins in over 100 individuals with ASD revealed reduced levels of IgG and immunoglobulin M (IgM) that inversely correlated with scores on the Aberrant Behavior Checklist (ABC), with lethargy being especially pronounced in children with the lowest IgG [103]. The mean IgG level in ASD individuals equaled 5.39 mg/mL in contrast to 7.72 mg/mL in typically developing co6ntrols, and 8.23 mg/mL in children with developmental delay. IgM level was less evident; however, it was statistically significant (0.67 mg/mL in comparison to 0.79 mg/mL in healthy controls).

Further in-depth studies revealed that the detected abnormalities were not a result of B-cell dysfunction. There were no differences in the number of naïve, memory IgG or IgM cells, no abnormalities in response to antigenic stimulation, and production of immunoglobulins after in vitro stimulation was detected. The authors hypothesized that the lower level of immunoglobulins is caused by either a defect in another immune cell type that takes part in immunoglobulin production or a defect during immune system development [104].

Plasma concentration of IgM as well as IgG, especially IgG4, was reported to be increased in ASD patients in comparison to healthy siblings. Moreover, IgG1 subclass was found to be increased in comparison with healthy siblings of the same gender [105]. Higher concentrations of IgA, IgG, and IgE food-specific antibodies in individuals with ASD in comparison to their siblings were also described [106]. Spiroski et al.’s study [107] of immunoglobulins in Macedonian children with ASD revealed alterations in concentration of IgA, IgG2, and IgG3 between ASD children and healthy family members. The study was a retrospective analysis that included 30 autistic children with mean age of 10; the control groups consisted of mothers, fathers, and siblings of affected children. No exact demographical data was given. Because of multiple comparison testing and lack of precise characteristics of examined subjects, study results should be interpreted with caution.

According to Croonenberghs et al. [108], autistic individuals had higher concentrations of total serum proteins with increased concentrations of albumin and gamma-globulins, especially IgG2 (305 md/dL vs. 216 md/dL) and IgG4 (76 md/dL vs. 48 md/dL). The authors hypothesize that alteration of IgG subclasses may be associated with a cytokine-related influence on autoimmune B cells. Unfortunately, the study was carried out on a small number of subjects (18 with ASD and 22 controls). However, increased IgG4 levels in ASD children in comparison to typically developing controls and children with non-autistic developmental delay were confirmed in another study [109].

A higher frequency of D8/17 B lymphocytes was found in ASD subjects, especially in subjects with repetitive behaviors [110]. This antigen is associated with Tourette syndrome, rheumatic fever, and pediatric autoimmune neuropsychiatric disorders associated with streptococcal infections [111–114]. B cells were found to be hypersensitive to thimerosal [115]. Wei et al.’s study [116] on B-lymphocyte function in eight ASD individuals found altered cell migration and adhesion as well as improper immunoglobulin formation and secretion. The authors concluded that B-cell abnormalities were caused by disturbed integrin-FAK-Src signaling and reduced paxillin activity. However, the majority of studies assessing B-cell number and function did not detect any abnormalities [79, 80, 85, 117].

Overall data is suggestive that altered lymphocyte function, especially T-cell subpopulations, occurs in a substantial number of ASD individuals. Multiple studies have reported on lymphocyte pathology and imbalances between lymphocyte subpopulations. The key consistent findings include decreased response to stimulation [78–80], abnormal activation [80, 84, 85], improper ratio of T helper and suppressor cells [79, 80, 83], down-regulation of Th cells [81], and systemic deficit of Tregs [86, 87] in ASD subjects. These results, along with cytokine abnormalities, provide a broader view of a possible basis for observed ASD aberrations.

## Natural killer cells

Natural killer (NK) cells constitute about 15% of circulating lymphocytes and play a pivotal role in the innate immune system [118]. They are characterized by a lack of CD3 surface antigen, expression of CD56, and their function is exerted by production of immunomodulatory cytokines such as IFN-γ, tumor necrosis factor-alpha (TNF-α), and IL-10. They also have cytolytic activity and mediate cellular cytotoxicity and surveillance immune function through crosstalk with dendritic cells [119, 120]. Imbalances between their activation and inhibitory states could play a role in autoimmune diseases; however, the specific underlying mechanisms are not yet fully understood [121]. Roles for NK cells have already been described in the pathogenesis of neurological disorders such as multiple sclerosis [122], schizophrenia [123], Tourette syndrome [124], and Rett syndrome [125].

A previously described study by Ashwood et al. [82] reported higher absolute numbers of NK cells in peripheral blood of autistic individuals (161 cells/μL vs. 117 cells/μL). Reduced NK cell activity associated with ASD was first reported by Warren et al. in 1987 [126]. The study was carried out using K562 tumor cells, from 31 patients with ASD, as target cells and found that cells from 12 of the ASD patients had significantly reduced cytotoxic activity. Enstrom et al.’s study [127] on NK cells revealed an increased expression of NK cell receptor RNA, along with an up-regulated number of NK cells (21.24 ± 3.40 × 10^4^cells/mL vs. 14.45 ± 1.98 × 10^4^ cells/mL), and production of perforin, granzyme B, and IFN-γ in blood samples from ASD children compared to typically developing controls. Cellular studies were carried out on 17 autistic individuals aged 2–5 years, and 16 age- and sex-matched controls, while gene expression profile was conducted on 35 ASD children and 11 healthy controls. Cytotoxicity in response to stimulation was reduced in the ASD group. Similar abnormalities were not found in typically developing children. No differences in frequency of CD56^Dim^ (cytotoxic properties) or CD56^Bright^ (interleukin-producing cells) cells were noted. These results are concordant with previous studies that reported abnormalities in NK cell activity [126, 128] and molecular changes in differentially expressed genes [129]. According to Vojdani et al. [128], who analyzed peripheral blood samples from over 1000 children, NK cell activity is decreased due to low levels of its stimulants, IL-2 and IL-15. They found no correlation between NK cell absolute number and cytotoxic activity, in opposition to the previously mentioned hypothesis. However, the investigators found a relationship between cell function and low intracellular level of glutathione. They also showed that NK cell activity was higher after co-culture with glutathione, IL-2, and IL-15. However, serum concentrations of those interleukins and subpopulations of CD56^Dim^ and CD56^Bright^ were not assessed. In 59 adult ASD patients, significantly lower numbers of NKT cells, with normal numbers of NK cells, and increased numbers of stimulated NKT and NK cells expressing CD25^+^ were described [90].

Another approach for NK cell analysis was proposed by Torres et al. [130] and Guerini et al. [131] who focused on molecular changes by studying killer immunoglobulin receptors (KIR), MHC I class-binding receptors, that are expressed on NK cell surfaces and modulate NK cell function [132]. Both activation and inhibition states of KIR have been associated with various diseases including autoimmune disorders [133]. Torres et al. [130] found up-regulation of KIR-activating genes in patients with ASD. Another study [131] suggested that KIR might play a role in in utero ASD pathogenesis as activating KIR/HLA complexes were found to be increased in ASD, detectable in both children with ASD and their mothers.

The implications of NK cell pathology in ASD remain unknown, and they seem to be a secondary cause of proinflammatory processes and immune imbalances. Chronic inflammation at the cellular level could lead to persistent activation of NK cells, subsequent reduction of their cytotoxic activity, and compensatory up-regulation of their total number. Increased numbers of NK cells associated with ASD was confirmed in two [82, 127] out of five studies [82, 126–128, 134]. One study demonstrated the opposite result; however, the study and control group were broader in age demographics (respectively from 3 to 22 years old and from 1 to 12 years old). Abnormal NK cell functional activity was confirmed in both experiments that utilized cytotoxic ability analysis [127, 128]. One might suspect that either unnaturally low levels of NK cell stimulants (such as IL-15, IL-2, IL-12, IL-18, and IL-21) or that excessively high levels of the stimulants would be observed if the inflammatory milieu leads to NK cell pathogenic activation and loss of cytotoxic properties through exhaustion. Interestingly, abnormalities of these cytokines were scarcely, if ever, found. A major NK cell stimulant, IL-15, was explored in 7 of 57 conducted studies [135–141] and found significantly up-regulated only in children with ASD who presented with gastrointestinal (GI) disturbances [137]. IL-2 was more well studied [88, 91, 135, 136, 138–149] and turned out to be significantly altered in 4 out of 16 experiments. It was found to be up-regulated in comparison to control group in two studies [91, 149] and in one study it was increased among ASD children with GI issues and correlated with impaired adaptive behavior [138]. CD-4^+^ IL-2-secreting cells were found decreased in one of the earliest studies [88]. IL-12 was vastly explored in nearly half of the conducted studies [73, 91, 135, 136, 138–142, 144, 145, 147, 150–160] and found frequently increased in ASD subjects in comparison to healthy controls [135, 140, 141, 145, 152, 157, 160]. Interestingly, stimulation in different conditions down-regulated IL-12 concentration [144, 153, 154] apart from stimulation with dietary proteins [157]. IL-12 high concentration turned out to be associated with abnormal EEG [135], GI complaints [158], low IQ [138], and prominent aberrant behavior [144, 145]. IL-18 was explored in four studies [140–142, 159] and found elevated in adult males with ASD in comparison to neurotypical controls [140], while IL-21 was studied in one experiment and found significantly increased in autistic children compared to normal controls [162]. None of the studies assessed NK cell count and function in addition to cytokine levels.

## Monocytes

Abnormal monocyte count or function was described in ASD by several investigators [151, 155, 161]. Sweeten et al. [161] reported a high monocyte count in children with ASD, which was not confirmed in later studies. The difference was small (0.588 × 10^3^ cells/mm^3^ vs. 0.491 × 10^3^ cells/mm^3^), but statistically significant. Monocytes in children with ASD were also found to be positive for a surface receptor that is thought to be expressed on cells susceptible to apoptosis [90]. Enstrom et al. [155], in a small study on 17 ASD children and 16 healthy controls, stimulated monocytes with Toll-like receptor (TLR) ligands and observed differences between cytokine profiles in autistic and healthy children. Following different LTR stimulations, several cytokines were increased (IL-1β, IL-6, TNF-α) or decreased (IL-1β, IL-6, GM-CSF, and TNF-α) in ASD individuals. However, no difference in number of monocytes was observed. In children with ASD, stimulation with TLR2 and TLR4 led to a high proinflammatory response, while TLR9-induced stimulation resulted in poor production of cytokines and ineffective reaction. Authors concluded that such abnormalities may have an impact on neuronal activity and developing autoimmunity. Jyonouchi et al. [151] proposed an interesting study on monocyte cytokine profiles in six groups of patients with and without ASD, including an “inflammatory subtype of ASD” group—patients who presented with symptoms indicating immune dysregulation and GI complaints (other groups included ASD children without non-IgE-mediated food allergy, typically developing controls with non-IgE-mediated food allergy, children with pediatric acute-onset neuropsychiatric syndrome, and healthy controls). The proinflammatory cytokine profile in this group was up-regulated both before and after stimuli. Additionally, children who manifested irritability, lethargy, or hyperactivity were found to produce higher amounts of proinflammatory and lower amounts of anti-inflammatory cytokines (Tables 2 and 3). It is worth underlying that cytokine pattern in acute-onset neuropsychiatric syndrome was different than that observed in ASD.

**Table 2:** Concentration of cytokines, chemokines, and growth factors in peripheral blood of ASD patients: study participants’ description

Number	Study	Country	Study group age (years)	Groups	Females (%)	Medication (%)	ADHD (%)	Intellectual disability (%)	Epilepsy (%)	Psychological tools	Study material
1	Ahmad et al. 2017 [162]	Saudi Arabia	M: 7.69, SD: 2.26, R: 3–11	ASD (*n* = 50), HC (*n* = 45)	ASD (20), HC (22)	No	No data	No data	No	DSM-V, CARS	PBMNC, stimulation with PMA and ionomycin
2	Bryn et al. 2017 [142]	Norway	M: 11.2, SD: 2.02	ASD (*n* = 65) including ASD-C *n* = 30, ASD-A *n* = 12, ASD-AS *n* = 16, RS *n* = 1 and another ASD *n* = 6, HC (*n* = 30)	ASD (20), HC (53)	No data	Yes (18.5)	Yes (39)	Abnormal EEG (31)	ICD-10, ADI-R, ADOS, SCQ	Serum
3	Careaga et al. 2017 [150]	USA	Mdn: 3.21, IQR: 2.80–3.52	ASD (*n* = 50), HC (*n* = 16)	ASD (0), HC (0)	Not relevant	No data	No data	No	DSM-IV, ICD-10, ADOS, ADI-R, CBCL, MSEL, SCQ	Plasma, PBMNC supernatants, stimulation with PHA and LPS
4	Guloksuz et al. 2017 [163]	Turkey	M: 7.13, SD: 3.89	ASD (*n* = 28), PDD-NOS (*n* = 12), HC (*n* = 35)	ASD and PDD-NOS (25), HC (37)	No	No data	No data	No	DSM-IV, CARS	Plasma
5	Han et al. 2017 [164]	China	ASD-only—M: 9.38, SD: 2.84; ASD + ADHD—M: 9.67, SD: 2.18; HC—M: 10.92, SD: 3.95	ASD-only (*n* = 13), ASD + ADHD (*n* = 9), HC (*n* = 13)	ASD-only (0), ASD + ADHD (22), HC (31)	No immunosuppressive drugs	Yes (41)	None	No data	DSM-V, ADI-R, CCTT, SRS-2, WISC, CRS-R, FPT, Rey-O, TOL-DX	Serum
6	Jyonouchi et al. 2017^A^ [165]	USA	No data	ASD (*n* = 71)	No data	No data	No data	No data	No data	No data	Monocytes, stimulation with TLR agonists
7	Makinodan et al. 2017 [166]	Japan	M: 11.6, SD: 2.7	ASD (*n* = 30), HC (*n* = 30)	ASD (0), HC (0)	No psychoactive medications	No	No data	No	DSM-IV, ADI-R	PBMNC
8	El-Ansary et al. 2016 [73]	Saudi Arabia	R: 2–12	ASD (*n* = 35/29), HC (*n* = 38/16)	Gender-matched groups	No data	No data	No data	No	ADOS, ADI-R, 3DI, SSP	Plasma
9	Ferguson et al. 2016 [167]	USA	M: 11.8, SD: 3.8, R: 6–18	ASD (*n* = 120)	ASD (10)	No data	No data	Yes	No data	DSM-IV, ADOS, ABC, WISC/SB, VABS	Serum
10	Jácome et al. 2016 [135]	Cuba	M: 6.17, SD: 2.08, R: 3–9	ASD (*n* = 17), HC (*n* = 15)	ASD (29), HC (47)	No data	No data	No data	Yes (37.5), all ASD children had abnormal EEG	DSM-IV, CARS	Plasma
11	Pecorelli et al. 2016 [136]	Italy	M: 17.7, SD: 7.2, R: 9–37	ASD (*n* = 12), RTT (*n* = 10), HC (*n* = 8)	No data	No data	No data	No data	No data	DSM-V, ADOS, ABC	Serum
12	Rose et al. 2016^A^ [137]	USA	No data	ASD, HC	No data	No data	No data	No data	No data	No data	PBMNC, stimulation with TLR4 agonists
13	Akintunde et al. 2015 [94]	USA	M: 3.56, R: 2–5	ASD (*n* = 45), HC (*n* = 69)	ASD (18), HC (13)	No	No data	No data	No data	DSM-IV, ADI-R, ADOS,MSEL, SCQ, VABS	Plasma, PBMNC, stimulation with PHA
14	Barbosa et al. 2015 [168]	Brazil	M: 9.71, SD: 4.99	ASD (*n* = 30), HC (*n* = 18)	ASD (17), HC (17)	Risperidone (40), antidepressants (17), methylphenidate (17)	Yes	No data	No data	DSM-IV, SRS	Plasma
15	Tonhajzerova et al. 2015 [169]	Slovakia	M: 9.3, SD: 0.7	ASD (*n* = 15), HC (*n* = 20)	ASD (13)	No	No data	No data	No data	No data available	Plasma
16	Tsilioni et al. 2015 [170]	Greece	R: 4–10	ASD (*n* = 38), HC (*n* = 13)	ASD (15), HC (no data)	No psychoactive medications	No data	No data	No focal epilepsy	DSM, ADOS, VABS	Serum
17	Yang et al. 2015 [171]	China	M: 12.21, SD: 2.67	ASD (*n* = 35), HC (*n* = 31)	ASD (18), HC (22)	Not relevant	No	Yes (100)	No	DSM-IV, CARS	Plasma
18	El-Ansary et al. 2014 [172]	Saudi Arabia	R: 4–12	ASD (*n* = 20), HC (*n* = 19)	Gender-matched groups	No data	No data	No data	No	ADOS, ADI-R, 3DI	Plasma
19	Jyonouchi et al. 2014 [151]	USA	ASD-I—Mdn: 11.8, R: 6.0–27.0; ASD-NFA—Mdn: 7.5, R: 3.3–22, ASD-only—Mdn: 12.9, R: 3.6–20.5	ASD-I (*n* = 24), ASD-NFA (*n* = 20), ASD-only (*n* = 20), HC-NFA (*n* = 16), PANS (*n* = 18), HC (*n* = 16)	ASD-I (21), ASD-NFA (10), ASD-only (15), HC-NFA (44), PANS (10), HC (37)	Yes, no exact data	No data	Yes, no exact data	Yes (11)	ADOS, ADI-R, ABC, CSHQ, NCCPC, VABS	Monocytes, stimulation with TLR agonists
20	Al-Ayadhi et al. 2013 [89]	Saudi Arabia	M: 7.54, SD: 1.96, R: 4–12	ASD (*n* = 56), HC (*n* = 32)	ASD (18), HC (19)	No	No data	No data	No	DSM-IV, CARS	Serum
21	Napolioni et al. 2013 [138]	USA	M: 8.11, SD: 3.65	ASD (*n* = 25), HC (*n* = 25)	No data	No	No data	No exact data	No data	DSM-IV, ADI-R, ADOS, SB, SRS, VABS	Plasma
22	Ricci et al. 2013 [152]	Italy	R: 2–21	ASD (*n* = 29, including PDD-NOS *n* = 6), HC (*n* = 29)	ASD (6), HC (gender-matched)	Yes, no exact data	No	No exact data	No	DSM-IV, CARS	Serum
23	Al-Ayadhi et al. 2012 [93]	Saudi Arabia	M: 8.44, SD: 1.73, R: 6–11	ASD (*n* = 45), HC (*n* = 40)	ASD (20), HC (20)	No data	No data	No	No	DSM-IV, CARS	Serum
24	El-Ansary et al. 2012 [173]	Saudi Arabia	R: 3–16	ASD (*n* = 20), HC (*n* = 19)	ASD (0), HC (0)	No data	No data	No exact data	No	ADI-R, ADOS, 3DI	Plasma
25	Jyonouchi et al. 2012 [153]	USA	ASD-SPAD - Mdn:12.3, R:8.3-17.5, ASD-only - Mdn:8.1, R:5-17	ASD-SPAD (*n* = 8 including PDD-NOS *n* = 2), ASD-only (*n* = 39), HC (*n* = 37), HC-SPAD (*n* = 12)	ASD-SPAD (25), ASD-only (10), HC (22), HC-SPAD (50)	IVIG (all ASD-SPAD), multiple medications including antiepileptics	no data	no data	ASD-SPAD (50)	ADI-R, ADOS	PBMNC, cell culture supernatant, stimulation with ConA, PHA, recall Ag, IFN-γ-inducing cytokines
26	Manzardo et al. 2012 [139]	USA	Males—M: 7.82, SD: 1.47; females—M: 7.72, SD: 1.85	ASD (*n* = 99), unrelated SIB (*n* = 40)	ASD (25), unrelated SIB (30)	No data	No data	No data	No data	ADI-R, ADOS, SRS	Plasma
27	Onore et al. 2012 [175]	USA	Mdn: 2.88, IQR: 2.66–3.14	ASD (*n* = 49), HC (31)	ASD (14), HC (35)	No data	No data	No data	No data	ADI-R, ADOS, MSEL, SCQ	Plasma
28	Tostes et al. 2012 [143]	Brazil	No data available	ASD (*n* = 24), HC (*n* = 24)	No data available	No data available	No data available	No data available	No data available	DSM-IV	Plasma
29	Ashwood et al. 2011 [144]	USA	Mdn: 3.8, IQR: 3.2–4.3	ASD (66), HC (73)	ASD (11), HC (30)	No	No data	No data	No data	DSM-IV, ADI-R, ADOS, ABC, MSEL, SCQ, VABS	PBMNC, stimulation with PHA, tetanus toxoid, cell culture supernatants
30	Ashwood et al. 2011[174]	USA	ASD—Mdn: 3.6, IQR: 3.0–4.5; DD—Mdn: 3.5, IQR: 3.0–4.0	ASD (80), DD (27), HC (58)	ASD (16), DD (27), HC (33)	No	No data	No data	No data	DSM-IV, ADI-R, ADOS, ABC, MSEL, SCQ, VABS	Plasma
31	Ashwood et al. 2011 [145]	USA	ASD—Mdn:3.4, IQR: 2.9–4.3; DD—Mdn: 3.5, IQR: 3.0–4.1	ASD (*n* = 97), DD (*n* = 39), HC (*n* = 87)	ASD (13), DD (28), HC (18)	No	No data	No data	No data	DSM-IV, ADI-R, ADOS, ABC, MSEL, SCQ, VABS	Plasma
32	El-Ansary et al. 2011 [165]	Saudi Arabia	R: 4–12	ASD (*n* = 25), HC (*n* = 16)	ASD (0), HC (0)	No	No exact data	No data	No	ADI-R, ADOS	Plasma
33	Jyonouchi et al. 2011 [154]	USA	ASD-I—Mdn: 7.5, R: 3.0–15.6; ASD-only—Mdn: 5.9, R: 3.0–17.9	ASD-I (*n* = 30), ASD (*n* = 28), HC (*n* = 26)	ASD-I (10), ASD (*n* = 21), HC (*n* = 14)	No data	No data	No data	Yes (5)	DSM-IV, ADI-R, ADOS	PBMNC, stimulation with TLR agonists, T-cell mitogens, luminal Ag
34	Malik et al. 2011 [146]	USA	M: 8.4, SD: 0.27	ASD (*n* = 6), HC (*n* = 6)	No data	No data	No exact data	No data	No	DSM-IV, ADI-R	PBMNC
35	Schwarz et al. 2011 [140]	USA	M: 31.8, SD: 8.7	ASD (*n* = 45, only ASD-AS), HC (*n* = 50)	ASD (51), HC (48)	No data	No data	No data	No data	DSM-IV, AQ, EQ, SQ-R, WISC	Serum
36	Suzuki et al. 2011 [141]	Japan	M: 12.1, SD: 3.3, R: 7–15	ASD-HF (*n* = 28, including PDD-NOS *n* = 7), HC (*n* = 28)	ASD-HF (0), HC (0)	Not relevant	No exact data	No	No	DSM-IV, ADI-R, WISC	Plasma
37	Emanuele et al. 2010 [176]	Italy	M: 28.1, SD: 7.7, R: 18–44	Severe ASD (*n* = 22), HC (*n* = 28)	Severe ASD (18), HC (25)	No	No data	No exact data	No data	DSM-IV, ADI-R, AQ, CARS, RPM, VABS	Serum
38	Enstrom et al. 2010 [155]	USA	M: 3.9, R: 2.2–5.0	ASD (*n* = 17), HC (*n* = 16)	ASD (18), HC (19)	No immunomodulatory or antipsychotic drugs	No data	No data	No data	DSM-IV, ADI-R, ADOS, ABC, MSEL, SCQ, VABS	Monocytes, stimulation with TLR, cell culture supernatants
39	Kajizuka et al. 2010 [185]	Japan	M: 12.3, SD: 3.2, R: 6–19	ASD (*n* = 31), HC (*n* = 16)	ASD (0), HC (0)	No relevant	No exact data	No data	No	DSM-IV, ADI-R, WAIS	Serum
40	Ashwood et al. 2009 [147]	USA	Mdn: 3.42, R: 2.42–5	ASD (*n* = 18), HC (*n* = 19)	ASD (17), HC (5)	No antibiotics or anti-inflammatory drugs	No data	No data	No data	ADOS, ADI-R, SCQ	PBMNC, pretreatment with BDE-47, stimulation with LPS
41	Onore et al. 2009 [96]	USA	PHA stimulation—Mdn: 3.83, IQR: 3.17–4.25; PMA stimulation—Mdn: 4.25, IQR: 3.08–4.07	ASD (*n* = 34), HC (*n* = 26)	ASD (15), HC (19)	No data	No data	No data	No data	DSM-IV, ADI-R, ADOS, ABC, MSEL, SCQ, VABS	PBMNC, stimulation with PHA/PMA
42	Saresella et al. 2009 [91]	Italy	ASD—Mdn: 13, R: 5–17; SIB—Mdn: 15; R: 3–16	ASD (*n* = 20), SIB (*n* = 15), HC (*n* = 20)	ASD (*n* = 30), SIB (16), HC (45)	Not relevant	No data	No data	No data	DSM-IV, SRS	PBMNC, stimulation with staphylococcal enterotoxin B, IL-2
43	Ashwood et al. 2008 [177]	USA	ASD—Mdn: 3.4, IQR: 3.0–4.2; DD—Mdn: 3.9, IQR: 3.1–4.5	ASD (*n* = 75), DD (*n* = 32), HC (*n* = 36)	ASD (9), DD (12)	No data	No data	No data	No data	DSM-IV, ADI-R, ADOS, ABC, MSEL, SCQ, VABS	Plasma
44	Enstrom et al. 2008 [178]	USA	M: 3.5, R: 2–5	ASD (*n* = 40), HC (*n* = 20)	ASD (10), HC (20)	No data	No data	No data	No data	ADI-R, ADOS, MSEL, SCQ, MSEL, VABS	Plasma
45	Grigorenko et al. 2008 [179]	Netherlands	No exact data	ASD (*n* = 10/29 including ASD-AS, PDD-NOS and CDD), SIB (*n* = 10)	No exact data	No data	No data	No data	No data	ADOS, ADI	Plasma
46	Jyonouchi et al. 2008 [156]	USA	ASD-I—Mdn: 7.6, R: 2.3–13.4; ASD-only—Mdn: 4.8, R: 1.5–17.3	ASD-I (*n* = 26), ASD (*n* = 107), HC-FA (*n* = 24), HC (*n* = 43)	ASD-I (4), ASD (14), HC-FA (28), HC (28)	No data	No data	No data	Yes (4.5)	DSM-IV, ADOS, ADI-R	PBMNC, stimulation with TLR agonists
47	Molloy et al. 2006 [148]	USA	M: 6.9, SD: 2.0, R: 3.7–10.7	ASD (*n* = 20), HC (*n* = 20)	ASD (15), HC (15)	Psychotropic medications (65)	No data	No data	No data	DSM-IV, ADOS	PBMNC, stimulation with PHA, house dust mite, tetanus toxoid
48	Al-Ayadhi 2005 [180]	Saudi Arabia	M: 8.8, SD: 0.5, R: 3.5–14	ASD (*n* = 77, including ADD *n* = 8, RTT *n* = 2, ASD-AS *n* = 2), HC (*n* = 77)	ASD (8), HC (gender-matched)	No data	No data	No data	No exact data	E2	Serum
49	Jyonouchi et al. 2005 [157]	USA	ASD-GI—M: 4.7, R: 1.8–10.6, ASD-only—M: 5.4, R: 2.1–10.2, NFH—M: 2.8, R: 1.3–7.8	ASD-GI (*n* = 75, including PDD-NOS *n* = 27), ASD-only (*n* = 34, including PDD-NOS *n* = 1), NFH (*n* = 15), HC (*n* = 19)	ASD-GI (19), ASD-only (6), NFH (40), HC (42)	No exact data	No data	No data	No data	DSM-IV, ICD-10, ADI-R, ADOS	PBMNC, stimulation with gliadin, cow’s milk protein, soy, cell culture supernatants
50	Jyonouchi et al. 2005 [158]	USA	Mdn: 4.8	ASD (*n* = 177, including 77 on ED), NFH (*n* = 30, including 16 on ED), HC (*n* = 13)	No data	No data	No data	No data	No data	No data	PBMNC, stimulation with LPS
51	Sweeten et al. 2004 [181]	USA	M: 6.1, SD: 2.8, R: 2–12	ASD (*n* = 29), HC (*n* = 27)	ASD (14), HC (14)	No	No data	No data	No data	DSM-IV, ADI-R, ADOS	Plasma
52	Croonenberghs et al. 2002 [182]	Netherlands	R: 12–18	ASD (*n* = 13), HC (*n* = 13)	ASD (0)	Not relevant	No data	Yes (7)	No active seizure disorder	DSM-IV	Serum, whole blood culture supernatant
53	Jyonouchi et al. 2002 [183]	USA	Mdn: 5, R: 1–17	ASD (*n* = 72, including PDD-NOS *n* = 9, ASD-AS *n* = 1), DPI (*n* = 24), SIB (*n* = 26), HC (*n* = 15)	ASD (18), DPI (29), SIB (31), HC (20)	Valproic acid (4)	No data	No data	Yes (4)	DSM-IV, ICD-10, ADI-R, ADOS	PBMNC, stimulation with gliadin, cow’s milk protein, soy, cell culture supernatants
54	Jyonouchi et al. 2001 [159]	USA	Mdn: 6, R: 2–14	ASD (*n* = 71, including PDD-NOS *n* = 6), SIB (*n* = 23), HC (*n* = 17)	ASD (21), SIB (30), HC (59)	Valproic acid (7)	No data	No data	Yes (7)	DSM-IV	PBMNC, stimulation with LPS, PHA, tetanus, dust mite, IL-12p70, IL-18
55	Gupta et al. 1998 [88]	USA	R: 3–7	ASD (*n* = 20), HC (*n* = 20)	ASD (20), HC (15)	No data	No data	No data	No data	DSM-IV	PBMNC
56	Singh et al. 1996 [160]	USA	M: 10.7	ASD (*n* = 20), HC (*n* = 20)	ASD (20), HC (35)	Not relevant	No data	No data	No data	DSM-III	Plasma
57	Singh et al. 1991 [149]	USA	No data available	ASD, TD, DD	No data available	No data available	No data available	No data available	No data available	No data available	Serum

*M* mean, *SD* standard deviation, *R* range, *Mdn* median, *IQR* interquartile range, *ASD* autism spectrum disorder, *ADHD* attention deficit hyperactivity disorder, *RTT* Rett syndrome, *HC* healthy controls, *I* inflammatory subtype (defined as fluctuating behavioral symptoms following immune insults), *NFA* non-IgE-mediated food allergy, *SPAD* specific polysaccharide antibody deficiency, *LF* low functioning, *HF* high functioning, *DD* developmental delay, *PHA* phytohemagglutinin, *PMA* phorbol myristate acetate, *SIB* siblings, *GI* gastrointestinal symptoms, *PDD-NOS* pervasive developmental disorder not otherwise specified, *PANS* pediatric acute-onset neuropsychiatric syndrome, *FA* food allergy, *NHF* non-allergic food hypersensitivity, *ASD-C* childhood autism, *ASD-A* atypical autism, *ASD-AS* Asperger syndrome, *ADD* attention deficit disorder, *ED* elimination diet, *DPI* dietary protein intolerance, *CDD* childhood disintegrative disorder, *IVIG* intravenous immunoglobulins, *DSM* Diagnostic and Statistical Manual of Mental Disorders, *CARS* Childhood Autism Rating Scale, *ICD* International Statistical Classification of Diseases and Related Health Problems, *ADI-R* Autism Diagnostic Interview–Revised, *ADOS* Autism Diagnostic Observation Schedule, *SCQ* Social Communication Questionnaire, *CBCL* Child Behaviour Checklist, *MSEL* Mullen Scales of Early Learning, *CCTT* Children’s Color Trail Test, *CRS-R* Conners’ Rating Scales–Revised, *FPT* Five Point Test, *Rey-O* Rey–Osterrieth Complex Figure Test, *TOL-DX* Tower of London Test–Drexel Version, *3DI* Developmental, Dimensional Diagnostic Interview, *SSP* Short Sensory Profile, *ABC* Aberrant Behavior Checklist, *WISC* Wechsler Intelligence Scale for Children, *SB* Stanford–Binet, *VABS* Vineland Adaptive Behavior Scale, *SRS* Social Responsiveness Scale, *CSHQ* Children’s Sleep Habits Questionnaires, *NCCPC* Non-communicating Children’s Pain Checklist, *RBSR* Repetitive Behavior Scale–Revised, *AQ* Autism-Spectrum Quotient, *EG* Empathy Quotient, *SQ-R* Systemizing Quotient–Revised, *RPM* Raven’s Progressive Matrices, *WAIS* Wechsler Adult Intelligence Scale, *ADI* Autism Diagnostic Interview, *E2* Diagnostic Checklist Form E-2, *LIPS* Leiter International Performance Scale, *YSR* Youth Self-Report, *PBMNC* peripheral blood mononuclear cells, *LPS* lipopolysaccharides, *TLR* Toll-like receptors, *ConA* concanavalin A, *Ag* antigen, *IFN* interferon, *BDE-47* 2,2′,4,4′-tetrabromodiphenyl ether, *IL* interleukin

^A^Abstract

**Table 3:** Concentration of cytokines, chemokines, and growth factors in peripheral blood of ASD patients: immune abnormalities description

Number	Study	Cytokines	Chemokines	Growth factors	Other analytes	Main results	Relation to psychological symptoms	Excluded correlations
1	Ahmad et al. 2017 [162]	IL-21, IL-22, IL-27	x	x	CTLA-4 (CD152)	ASD vs. HC: ↑IL-21 and IL-22 CD4^+^ cells and mRNA expression, ↓IL-27 CD14^+^ cells and CTLA-4 CD4^+^ cells and mRNA	Not studied	x
2	Bryn et al. 2017 [142]	IL-1RA, IL-1β, IL-2, IL-6, IL-9, IL-10, IL-12, IL-18, IFN-γ, TNF-α	CXCL1 (GRO-α), CXCL8 (IL-8)	x	x	ASD-C vs. HC: ↑IL-8, ↓IL-10; ASD-C vs. ASD-AS: ↑IL-8	Not studied	No differences between ASD and HC children
3	Careaga et al. 2017 [150]	IL-1β, IL-6, IL-10, IL-12p40, IL-13, IL-17, IFN-γ, TNF-α	CCL2 (MCP-1)	GM-CSF	x	ASD children divided into 2 groups according to the response to LPS stimulation, ASD-high vs. ASD-low: ↑IL-1β, IL-6, IL-10, MCP-1	Social and developmental impairment, sleep disturbances, aggression in ASD-high group: ↑IL-1β, IL-6, IL-10, MCP-1; developmental impairment more severe in children with Th1-skewed response to stimulation	x
4	Guloksuz et al. 2017 [163]	IL-1β, IL-4, IL-6, IL-10, IL-17A, IFN-γ, TNF-α	x	x	S100B	ASD vs. HC: ↑S100B, TNF-α	Severe ASD: ↑S100B	No association with regression, no association of S100B concentration and ASD severity after adjustment for age, gender, and BMI
5	Han et al. 2017 [164]	TGF-β1, MIF	CCL2 (MCP-1), CCL5 (RANTES), CXCL8 (IL-8), CXCL-9 (MIG), CXCL10	x	x	ASD vs. HC: ↑CCL2 and CCL5 ↓CXCL9; ASD + ADHD vs. ASD-only: ↑MIF ↓CXCL8	Poor executive functioning: ↑MIF ↓CXCL10; impaired short-term memory: ↑CXCL9; severe inattention and hyperactivity: ↓CXCL5	No association with social domain and repetitive, restricted behaviors and interests
6	Jyonouchi et al. 2017^A^ [184]	IL-1β, IL-10	x	x	Oxygen consumption rate in PBMNC	Altered IL-1β and mitochondrial function may be associated with chronic GI symptoms	No data	x
7	Makinodan et al. 2017 [166]	IL-1β, IL-6, TNF-α (mRNA levels)	x	x	x	ASD vs. HC: ↓TNF-α	Impaired social interactions: ↓TNF-α	No association with impairment of communication, restricted behaviors, and interests
8	El-Ansary et al. 2016 [73]	IL-10, IL-12	x	x	NF-κB, 8-isoprostane, PE, PS, PC, MAP2K1, PGE2, PGE2-EP2, mPGES-1, cPLA2, COX-2	Biomarker sets effective in distinguishing ASD from HC subjects	Sensory impairment: PE, PGES, mPGES-1	No association with cognitive and social impairment
9	Ferguson et al. 2016 [167]	IL-6, TNF-α	x	x	Salivary cortisol	ASD with more prominent upper GI symptoms: ↑TNF-α	Higher IQ: ↓IL-6, socialization impairment: ↑IL-6, irritability: ↓TNF-α, anxiety: ↑TNF-α, regression: ↑TNF-α	No association of IL-6 with GI symptoms, no correlation with depression and seizures
10	Jácome et al. 2016 [135]	IL-1α, IL-1RA, IL-1β, IL-2, IL-4, IL-5, IL-6, IL-6sR, IL-7, IL-10, IL-12p40, IL-12p70, IL-13, IL-15, IL-16, IL-17, IFN-γ, TNF-α, TNF-β, TNF-sRI, TNF-sRII	CCL1 (I-309), CCL2 (MCP-1), CCL3 (MIP-1α), CCL4 (MIP-1β), CCL5 (RANTES), CCL9 (MIP-1γ), CCL11 (eotaxin), CCL24 (eotaxin-2), CXCL8 (IL-8), CXCL-9 (MIG), CXCL13 (BLC)	G-CSF, M-CSF, GM-CSF, PDGF-BB	ICAM-1, TIMP1, TIMP2	ASD vs. HC: ↑IL-1β, IL-6, IL-17, IL-12p40, and IL-12p70; mild ASD vs. HC: ↑IL-1β, IL-6, IL-12p40, IL-17; ASD + abnormal EEG: ↑IL-12p40 ↑IL-6	Moderate ASD vs. HC: ↑IL-1β, IL-6, IL-12p70, IL-17, TNF-α; moderate ASD vs. mild ASD: ↑IL-6, IL-12p70, TNF-α and ↓IL-12p40	x
11	Pecorelli et al. 2016 [136]	IL-1RA, IL-1β, IL-2, IL-4, IL-5, IL-6, IL-7, IL-9, IL-10, IL-12p70, IL-13, IL-15, IL-17, IFN-γ, TNF-α	CCL2 (MCP-1), CCL3 (MIP-1α), CCL4 (MIP-1β), CCL5 (RANTES), CCL11 (eotaxin), CXCL8 (IL-8), CXCL10 (IP-10)	G-CSF, GM-CSF, FGF, PDGF-BB, VEGF	x	ASD vs. RTT: ↓IL-1β, VEGF	Not studied	ASD vs. HC: no significant differences
12	Rose et al. 2016^A^ [137]	i.a. IL-1α, IL-1β, IL-5, IL-15, IL-17, TNF-α	x	x	x	ASD-only vs. HC: ↑IL-1α, IL-1β, TNF-α; ASD-GI vs. ASD-only: ↑IL-5, IL-15, IL-17, ↓TGF-β; ASD-GI vs. HC: ↓TGF-β	Worse score on ABC: ASD with GI symptoms	x
13	Akintunde et al. 2015 [94]	IL-4, IL-13, IL-17	x	x	x	ASD vs. HC after PHA: ↑IL-17; ASD + asthma vs. HC + asthma after PHA: ↑IL-17, IL-13	Not studied	No differences in baseline levels of cytokines between ASD and HC, no differences in IL-17 in children with and without asthma, no association with allergies
14	Barbosa et al. 2015 [168]	IL-1β, IL-33	x	x	sST2	Positive correlation of IL-33 and sST2	No correlations	No differences in baseline levels, no differences in relation to medications
15	Tonhajzerova et al. 2015 [169]	IL-1β, TNF-α,	CXCL8 (IL-8)	x	TBARS	ASD vs. HC: ↑IL-8	Not studied	X
16	Tsilioni et al. 2015 [170]	IL-6, IL-9, IL-31, IL-33, TNF	x	x	x	ASD vs. HC: ↑TNF, authors distinguished two subgroups of ASD children: with initially low or high IL-6 and TNF level	Not studied at baseline level of cytokines	No significant difference in baseline IL-6, IL-9, IL-31, IL-33
17	Yang et al. 2015 [171]	IL-6	x	x	5-HT	ASD vs. HC: ↑IL-6, 5-HT	ASD severity: ↑IL-6, 5-HT	x
18	El-Ansary et al. 2014 [172]	IL-6, IFN-γ, TNF-α	x	x	GABA, glutamate/GABA ratio, IFI-16	ASD vs. HC: ↑GABA, glutamate, IFN-γ, IFI-16; ↓glutamate/GABA ratio, IL-6, TNF-α	Not studied	x
19	Jyonouchi et al. 2014 [151]	IL-1β, IL-6, IL-10, IL-12p40, IL-17a, IL-23, TGF-β, TNF-α, sTNFRII	x	x	x	ASD-I-flare vs. ASD-NFA, ASD, HC: ↑IL-1β, IL-6	Worsening of irritability, lethargy, and hyperactivity in ASD-I: ↑IL-1β, IL-6 ↓IL-10	x
20	Al-Ayadhi et al. 2013 [89]	x	CCL17 (TARC), CCL22 (MDC)	x	x	ASD vs. HC: ↑TARC, MDC	Severe ASD vs. mild–moderate ASD: ↑TARC, MDC	x
21	Napolioni et al. 2013 [138]	IL-1α, IL-1RA, IL-1β, IL-2, IL-4, IL-5, IL-6, IL-6sR, IL-7, IL-10, IL-11, IL-12p40, IL-12p70, IL-13, IL-15, IL-16, IL-17, IFN-γ, TNF-α, TNF-β, TNF-sRI, TNF-sRII	CCL1 (I-309), CCL2 (MCP-1), CCL3 (MIP-1α), CCL4 (MIP-1β), CCL5 (RANTES), CCL11 (eotaxin), CCL15 (MIP-1δ), CCL24 (eotaxin-2), CXCL8 (IL-8), CXCL9 (MIG), CXCL13 (BLC)	G-CSF, M-CSF, GM-CSF, PDGF-BB	ICAM-1, TIMP-2	Head circumference: ↓BLC, TIMP-2; GI issues: ↑IL-1β, IL-2, IL-6	Regression: ↑IL-1β, IL-5, IL-17; non-verbal: ↑GM-CSF, M-CSF, IL-10; VABS score: ↓GM-CSF, IL-1β, IL-2, IL-6, MCP-1, ↑MIP-1δ; social responsiveness: ↓IL-6sR, MIP-1β, MIP-1δ; IQ: ↓IL-1β, IL-6, IL-7, IL-11, IL-12p70, IL-13, IL-16, IL-17, M-CSF, GM-CSF, TNF-sRII	No association with allergy
22	Ricci et al. 2013 [152]	IL-1β, IL-6, IL-12, IL-13, IL-23, TNF-α	x	BDNF	x	ASD vs. HC: ↑IL-1β, IL-6, IL-12, IL-23, TNF-α, BDNF	ASD severity: ↑IL-1β	No correlation with IL-13
23	Al-Ayadhi et al. 2012 [93]	IL-17A	x	x	x	ASD vs. HC: ↑IL-17A	ASD severity: ↑IL-17A	x
24	El-Ansary et al. 2012 [173]	IFN-γ, TGF-β2	x	x	HSP70, caspase 7	ASD vs. HC: ↑IFN-γ, TGF-β2, HSP70, caspase 7	Not studied	x
25	Jyonouchi et al. 2012 [153]	IL-1β, IL-6, IL-10, IL-12p40, IL-23, TGF-β, TNF-α, sTNFRII	x	x	Monocyte transcription profiling	ASD-SPAD vs. HC: ↓IL-1β (baseline), IL-6 (baseline, after TLR7/8, TLR2/6), IL-10 (after *Candida* antigen), IL-12p40 (after TLR4, T-cell mitogens, *Candida* antigen), IL-17 (after T-cell mitogens), IL-23 (after TLR7/8), TNF-α (baseline), IFN-γ (after T-cell mitogens). ASD-SPAD vs. ASD-only: ↑gene expression of TGFBR, Notch, EGFR1 pathways	Not studied	ASDnoSPAD vs. HC: no significant differences
26	Manzardo et al. 2012 [139]	IL-1α, IL-1β, IL1-RA, IL-2, IL-2RA, IL-3,IL-4, IL-5, IL-6, IL-7, IL-9, IL-10, IL-12p40, IL-12p70, IL-13, IL-15; IL-17, IFN-α2, IFN-γ, TGF-α, TNF-α, TNF-β	CCL2 (MCP-1), CCL3 (MIP-1α), CCL4 (MIP-1β), CCL7 (MCP-3), CCL11 (eotaxin), CCL22 (MDC), CXCL1 (GRO-α), CXCL8 (IL-8), CXCL10 (IP-10)	EGF, FGF-2, G-CSF, GM-CSF, VEGF	CD40L, Flt3 ligand	ASD vs. unrelated SIB: ↓IL-1α, IL-6, G-CSF, EGF, fractalkine, MCP3, MIP1, MIP1	Not studied	x
27	Onore et al. 2012 [175]	x	x	EGF, HGF	x	ASD vs. HC: ↓EGF	Not studied	No differences in HGF concentration
28	Tostes et al. 2012 [143]	IL-1β, IL-2, IL-4, IL-6, IL-10, IFN-γ, TNF-α	x	x	VIP, NT-3, NO	ASD vs. HC: ↑IFN-γ, VIP, NO ↓NT-3	Not studied	x
29	Ashwood et al. 2011 [144]	IL-1β, IL-2, IL-4, IL-5, IL-6, IL-10, IL-12p40, IL-13, IFN-γ, TNF-α	CXCL8 (IL-8)	GM-CSF	Lymphocyte subpopulations and markers of cellular activation CD134, CD25, CD69, CD95, HLA-DR	ASD vs. HC: high response to PHA stimulation ↑IL-8 (baseline), GM-CSF, TNF-α, IL-13 (PHA) ↓IL-12p40 (PHA), IFN-γ (tetanus toxoid); ↓CD134^+^ and CD25^+^ cells after PHA stimulation	Increased hyperactivity: high response to PHA stimulation, ↑IL-12p40, ↓IL-13; increased lethargy: high response to PHA stimulation; stereotypy: ↑TNF-α, IFN-γ, ↓GM-CSF; impaired communication: ↑IL-8, IFN-γ; inappropriate speech: ↑IL-12p40; impaired expressive language: ↓IL-5, IL-10; impaired fine motor skills: ↓IL-5; impaired visual reception: ↑IL-8; impaired expressive language: ↓IL-5; impaired adaptive behavior: ↓GM-CSF	No association with regression, no differences after tetanus toxoid stimulation, no differences in number of CD3^+^, CD4^+^ and CD8^+^ T cells, no differences in CD69^+^, CD137^+^, and HLA-DR^+^ cells
30	Ashwood et al. 2011 [174]	x	CCL2 (MCP-1), CCL3 (MIP-1α), CCL4 (MIP-1β), CCL5 (RANTES), CCL11 (eotaxin), CXCL10 (IP-10)	x	x	ASD vs. DD, HC: ↑MCP-1, RANTES, eotaxin; ASD vs. DD: ↑MIP-1β	Impaired communication: ↑MIP-1α, RANTES; impaired behaviors: ↑MCP-1, RANTES and eotaxin; impaired adaptive and cognitive functions: ↑MCP-1, RANTES, eotaxin; lethargy: ↑RANTES, eotaxin; hyperactivity: ↑RANTES, eotaxin; stereotypy: ↑RANTES, eotaxin; impaired visual reception: ↑MCP-1, RANTES, eotaxin; impaired fine motor skills: ↑MCP-1, RANTES, eotaxin; impaired expressive language: ↑MCP-1, RANTES, eotaxin; worse daily living scores: ↑MCP-1, eotaxin; impaired adaptive behavior: ↑RANTES, eotaxin	No differences in MIP-1α and IP10 concentrations
31	Ashwood et al. 2011 [145]	IL-1β, IL-2, IL-4, IL-5, IL-6, IL-10, IL-12p40, IL-13, IFN-γ, TNF-α	CXCL8 (IL-8)	GM-CSF	x	ASD vs. DD, HC: ↑IL-6, IL-12p40; ASD vs. HC: ↑IL-1β, IL-8; regressive ASD vs. early-onset ASD: ↑IL-1β, GM-CSF; regressive ASD vs. HC: ↑IL-1β, IL-6, IL-8, GM-CSF	Impaired non-verbal communication: ↑IL-4; stereotypies: ↑IL-1β, IL-6, IL-8, IL12p40; lethargy: ↑IL-8, IL-12; hyperactivity: ↑IL-8, impaired visual reception: ↑IL-8; impaired receptive and expressive language: ↑IL-8; impaired daily living: ↑IL-8	No association with IQ, no psychological differences in children with and without regression
32	El-Ansary et al. 2011 [165]	IL-6, TNF-α	x	x	Caspase 3	ASD vs. HC: ↓caspase3, IL-6, TNF-α	Not studied	x
33	Jyonouchi et al. 2011 [154]	IL-1β, IL-5, IL-6, IL-10, IL-12p40, IL-17A, IL-23, TGF-β, TNF-α, sTNFRII	x	x	Transcription profiling	ASD-I-GI vs. HC: ↓IL-1β, IL-6, IL-10; ASD-I-GI vs. HC after TLR stimulation: ↓IL-1β, IL-10, IL-12, IL-23, TNF-α; ASD-I-GI vs. HC after luminal Ags stimulation: ↓IFN-γ, TNF-α; 7 pts from ASD-I diagnosed with immunodeficiency: 1-CVID, 6-SPAD	Not studied	No differences between ASD-I without GI and HC apart from lower IL-23 production, no difference after stimulation with T-cell mitogens
34	Malik et al. 2011 [146]	IL-1, IL-2, IL-4, IL-5, IL-6, IL-10, IFN-γ, TNF-α	CXCL8 (IL-8)	GM-CSF	Bcl2 concentration and expression	ASD vs. HC: ↑IL-6, TNF-α, cathepsin D expression; ↓ Bcl2 expression	Not studied	No differences in concentration of Blc2, IL-1β, IL-2, IL-4, IL-5, IL-8, IL-10, IFN-γ, GM-CSF
35	Schwarz et al. 2011 [140]	IL-1α, IL-1β, IL-1RA, IL-2, IL-23, IL-3, IL-4, IL-5, IL-6, IL-7, IL-10, IL-11, IL-12p40, IL-12p70, IL-13, IL-15, IL-16, IL-17, IL-18, IFN-γ, MIF, TGF-α, TGF-β3, TNF-α, TNF-β, TNFRII	CCL1 (I-309), CCL2 (MCP-1), CCL3 (MIP-1α), CCL4 (MIP-1β), CCL5 (RANTES), CCL7 (MCP-3), CCL11 (eotaxin), CCL22 (MDC), CCL26 (eotaxin-3) CXCL1 (GRO-α), CXCL8 (IL-8)	EGF, EGF-R, HB-EGF, FGF basic, FGF-4, M-CSF, G-CSF, GM-CSF, HGF, NGF-β, SCF, IGF-I, IGF-BP, PDGF, VEGF	Multiple analytes including IgA, IgM, IgE, CD40, CD40L, ICAM-1, MMP-2, MMP-3, MMP-9, s100B, TIMP-1	Female ASD vs. HC: ↑IL-1β, IL-7, IL-12p40, NARG1, FAI, LH, TF, BDNF; ↓GOT1, Apo-CIII, IgM, sRAGE, Apo-A1, Tenascin-C, Eotaxin-3, Endothelin-1, GH, male ASD vs. HC: ↑IL-1β, IL-3, IL-4, IL-5, IL-10, IL-12p40, IL-12p70, IL-18, fatty acid binding protein, EPO, G-CSF, chromogranin A, neuronal cell adhesion molecule, tenascin-C, TNF-α, ENA-78, factor VII, connective tissue growth factor, thrombopoietin, stem cell factor, sortilin 1, ICAM-1 ↓GOT-1	Not studied	x
36	Suzuki et al. 2011 [141]	IL-1α, IL-2Rα, IL-1RA, IL-1β, IL-2, IL-3, IL-4, IL-5, IL-6, IL-7, IL-9, IL-10, IL-12p40, IL-12p70, IL-13, IL-15, IL-16, IL-17, IL-18, IFN-γ, LIF, TNF-α, TNF-β, TRAIL, SCF	CCL2 (MCP-1), CCL3 (MIP-1α), CCL4 (MIP-1β), CCL5 (RANTES), CCL7 (MCP-3), CCL11 (eotaxin), CXCL1 (GRO-α), CXCL8 (IL-8), CXCL9 (MIG), CXCL10 (IP-10), CXCL12 (SDF-1α), CTACK	HGF, M-CSF, G-CSF, GM-CSF, VEGF, basic FGF, PDGF-BB, β-NGF, SCGF-β	x	ASD vs. HC: ↑IL-1β, IL-1RA, IL-5, IL-8, IL-12p70, IL-13, IL-17, GRO-α	Not studied	x
37	Emanuele et al. 2010 [176]	IL-1β, IL-6, IL-10	x	x	Endotoxin, sCD14	ASD vs. HC: ↑endotoxins, IL-1β, IL-6	Impaired social interaction: ↑endotoxins	No differences in concentrations of sCD14 and IL-10, no association with IQ
38	Enstrom et al. 2010 [155]	IL-1β, IL-4, IL-5, IL-6, IL-10, IL-12p70, IFN-γ, TNF-α	CCL2 (MCP-1), CXCL8 (IL-8)	GM-CSF	Monocyte counts and subsets (CD14^+^CD16^+^ and CD14^+^CD15^−^), plasma	ASD vs. HC: ↑IL-1β (TLR2-LTA, TLR4-LPS), IL-6 (TLR2-LTA), TNF-α (TLR2-LTA), CD14^+^HLA-DR^+^ (baseline, TLR2-LTA) ↓MCP-1 (TLR4-LPS, TLR3-poly I:C, TLR9-CpG-B), IL-1β (TLR9-CpG-B), IL-6 (TLR9-CpG-B), TNF-α (TLR9-CpG-B), GM-CSF (TLR9-CpG-B)	Impaired social interaction: ↑IL-1β (TLR4-LPS), IL-6 (TLR-4-LPS); impaired non-verbal communication: ↑IL-1β (TLR4-LPS), IL-6 (TLR-4-LPS)	No differences in total number of monocytes and it subsets, no association with GI symptoms. TLR stimulation showed no association with Th1 (IL-12p70, IFN-γ) and Th2 (IL-4, IL-5, IL-10) cytokines in plasma
39	Kajizuka et al. 2010 [185]	x	x	PDGF-AA, PDGF-AB, PDGF-BB, VEGF	x	ASD vs. HC: ↑PDGF-BB	Restricted behaviors and interests: ↑PDGF-BB	No association with IQ, impairment of social interaction and communication
40	Ashwood et al. 2009 [147]	IL-1β, IL-2, IL-4, IL-5, IL-6, IL-10, IL-12p40, IFN-γ, TNF-α	CCL2 (MCP-1), CCL3 (MIP-1α), CCL4 (MIP-1β), CCL5 (RANTES), CCL11 (eotaxin), CXCL8 (IL-8)	GM-CSF	x	ASD vs. HC: ↑MIP-1β after pretreatment with BDE-47; ASD vs. HC: ↓IL-6, GM-CSF and ↑IL-1β, IL-8 after pretreatment with BDE-47 and stimulation with LPS	Not studied	Undetectable basal levels of IL-2, IL-4, IL-5, IFN-γ, MCP-1, RANTES, and eotaxin, pretreatment with BDE-47—no effect on cytokine production
41	Onore et al. 2009 [96]	IL-17, IL-23	x	x	x	ASD vs. HC: ↓IL-23	Impaired social interactions: ↓IL-23 after PHA stimulation	No differences in total numbers of T cells, B cells, or monocytes, undetectable levels of IL-17 and 23 without stimulation, no differences in IL-17 following stimulation with PHA, no differences in the frequency of Th17 cells, no association with ADI-R, MSEL, VABS, and ABC scores
42	Saresella et al. 2009 [91]	IL-1β, IL-2, IL-6, IL-10, IL-12, IFN-γ, TNF-α	x	x	Perforin, granzyme	ASD vs. HC: ↑CD4^+^IFN-γ, CD4^+^IL-6, CD4^+^IL-10, CD8^+^IFN-γ, CD8^+^IL-2, CD8^+^IL-6, CD8^+^IL-10, CD14^+^IL-10, ↓CD14^+^IL-6, CD14^+^IL-1β; ASD vs. SIB: ↑CD4^+^TNF-α, CD8^+^IFN-γ; SIB vs. HS: ↑CD4^+^IL-6, CD4^+^IL-10, CD8^+^IL-2, CD8^+^TNF-α, CD8^+^IL8, CD8^+^IL-10 ↓CD14^+^TNF-α; ASD and SIB vs. HC: ↑naive CD8^+^CD45RA-CCR7^+^ lymphocytes, ↓effector memory CD8^+^CD45RA-CCR7^−^ and terminally differentiated CD4^+^CD45RA^+^CCR7^−^ lymphocytes	Not studied	No statistically significant differences in granzyme and perofin-producing CD8^+^ lymphocytes, no differences in basic lymphocyte subpopulations
43	Ashwood et al. 2008 [177]	TGF-β1	x	x	x	ASD vs. HC: ↓TGF-β1, ASD vs. DD: ↓TGF-β1	Irritability, lethargy, stereotypy, and hyperactivity: ↓TGF-β1, impaired adaptive behavior, social interaction: ↓TGF-β1, especially in early-onset ASD; regressive ASD and irritability: ↓TGF-β1	No differences in concentration of TGF-β1 and psychological parameters within ASD regressive and early-onset subtypes, no correlation with ADI-R, ADOS, MSEL, or VABS scores
44	Enstrom et al. 2008 [178]	IL-17, IL-23	x	x	x	ASD vs. HC: ↓IL-23; early-onset ASD vs. HC: ↓IL-23; early-onset ASD vs. regressive ASD: ↓IL-23	Not studied	No significant difference in IL-17 concentration, no significant difference in IL-23 in regressive ASD vs. HC
45	Grigorenko et al. 2008 [179]	MIF	x	x	x	ASD vs. SIB: ↑MIF	ASD severity, social impairment, imaginative skills: ↑MIF	x
46	Jyonouchi et al. 2008 [156]	IL-1β, IL-6, IL-10, IL-12p40, IL-23, TGF-β, TNF-α, sTNFRII	x	x	x	ASD-I vs. ASD, HC : ↓IL-1β (TLR4/7/8), IL-10 (TLR2/6) ↑IL-23 (TLR4)	Not studied	x
47	Molloy et al. 2006 [148]	IL-2, IL-4, IL-5, IL-10, IL-13, IFN-γ	x	x	Eosinophil count	ASD vs. HC: ↑IL-4, IL-5, IL-13, IL-13/IL-10 ratio, IFN-γ/IL-10 ratio, eosinophil count; ASD without medication vs. TD: ASD vs. TD: ↑IL-4, IL-13	Not studied	No differences in IL-10 baseline concentration, no significant differences between ASD and HC following stimulation, no differences in cytokine concentration in relation to medication
48	Al-Ayadhi 2005 [180]	IL-1, IL-6, TNF-α	x	x	x	ASD vs. HC: ↑TNF-α, IL-1, IL-6	No correlations with ASD severity	x
49	Jyonouchi et al. 2005 [157]	IL-5, IL-10, IL-12p40, IFN-γ, TNF-α, sTNFRII	x	x	x	ASD vs. HC after stimulation: ↑TNF-α, IL-12; NFH vs. HC: ↑IFN-γ, TNF-α, IL-10, IL-12	Not studied	No differences in IL-5 concentration after stimulation, no significant differences between groups without stimulation
50	Jyonouchi et al. 2005 [158]	IL-1RA, IL-1β, IL-6, IL-10, IL-12, TNF-α, sTNFRII	x	x	x	ASD vs. HC: ↑TNF-α (LPS); ASD-GI without diet vs. ASD: ↑IL-12 (LPS), ↓IL-10 (LPS)	Not studied	No significant difference in cytokine production after T-cell mitogens
51	Sweeten et al. 2004 [181]	IL-1β, IFN-y, TNF-α	x	x	NO	ASD vs. HC: ↑NO	Not studied	No differences in IL-1β, IFN-y, TNF-α concentrations between study and control groups
52	Croonenberghs et al. 2002 [182]	IL-1RA, IL-2R (serum only), IL-6, IL-10 (supernatant), IFN-γ (supernatant), TNF-α (supernatant)	x	x	x	ASD vs. HC: ↑IL-1RA, IFN-γ (culture supernatants)	Not studied	No differences in serum cytokine concentrations
53	Jyonouchi et al. 2002 [183]	IL-1β, IL-4, IL-5, IL-6, IFN-γ, TNF-α, sTNFRI, sTNFRII	x	x	x	ASD vs. HC without stimulation: ↑ IL-5; ASD vs. HC after gliadin or milk stimulation: ↑ IFN-γ, TNF-α; ASD vs. HC after soy stimulation: ↑IFN-γ; SIB vs. HC after milk stimulation: ↑IFN-γ, TNF-α; DPI vs. HC after stimulation: ↑IFN-γ, TNF-α	Not studied	No differences in IL-5 concentration after stimulation; differences between ASD, SIB and DPI children not studied
54	Jyonouchi et al. 2001 [159]	IL-1RA, IL-1β, IL-4, IL-5, IL-6, IL-10, IL-12p40, IL-18, IFN-γ, TGF-β, TNF-α, sTNFRI, sTNFRII	x	x	x	ASD vs. HC: ↑IL-1β, IL-6 (only without stimulation), TNF-α, sTNFRI, sTNFRII (only without stimulation), ASD vs. SIB: ↑TNF-α, sTNFRI (only without stimulation)	Not studied	No differences in IL-1RA concentrations between groups, no differences in IL-1β, IL-6, and sTNFRII between ASD children and their healthy siblings
55	Gupta et al. 1998 [88]	IL-2, IL-4, IL-6, IL-10, IFN-γ	x	x	x	ASD vs. HC: ↑IL-4^+^CD4^+^, IL-4^+^CD8^+^, ↓IFN-γ^+^CD4^+^, IL-2^+^CD4^+^, IFN-γ^+^CD8^+^, IL-2^+^CD8^+^	Not studied	x
56	Singh et al. 1996 [160]	IL-6, IL-12, IFN-α, IFN-γ, TNF-α	x	x	sICAM-1	ASD vs. HC: ↑IL-12, IFN-γ	Not studied	No differences in concentrations of IFN-α, IL-6, TNF-α, sICAM-1
57	Singh et al. 1991 [149]	IL-1, IL-2, IL-2R	x	x	T8 antigen	ASD vs. HC, DD: ↑IL-2, T8 antigen	Not studied	No differences in concentrations of IL-1 and IL-2R

*IL* interleukin, *IFN* interferon, *TNF* tumor necrosis factor, *TGF* transforming growth factor, *MIF* macrophage migration inhibitory factor, *sR* soluble receptor, *TRAIL* TNF-related apoptosis-inducing ligand, *LIF* leukemia inhibitory factor, *SCF* stem cell factor, *CXCL* C-X-C motif chemokine ligand, *GRO* growth-regulated oncogene, *MCP* monocyte chemoattractant protein, *CCL* C-C motif chemokine ligand, *RANTES* regulated on activation, normal T-cell expressed and secreted, *I-309* T-lymphocyte activation, *MIP* macrophage inflammatory protein, *MIG* monokine induced by interferon-gamma, *BCL* B lymphocyte chemoattractant *TARC* thymus and activation-regulated chemokine, *MDC* macrophage-derived chemokine, *IP-10* IFN-γ-inducible protein 10, *SDF* stromal cell-derived factor, *CTACK* cutaneous T-cell-attracting chemokine, *GM-CSF* granulocyte-macrophage colony-stimulating factor, *G-CSF* granulocyte colony-stimulating factor, *M-CSF* macrophage colony-stimulating factor, *PDGF* platelet-derived growth factor, *FGF* fibroblast growth factor, *VEGF* vascular endothelial growth factor, *BDNF* brain-derived neurotrophic factor, *EGF* epidermal growth factor, *HGF* hepatocyte growth factor, *EGF-R* epidermal growth factor receptor, *HB-EGF* heparin-binding epidermal growth factor, *NGF* nerve growth factor, *IGF* insulin-like growth factor, *BP* binding protein, *SCGF* stem cell growth factor, *CTLA* cytotoxic T-lymphocyte-associated protein, *S100B* calcium-binding protein B, *PBMNC* peripheral blood mononuclear cells*, NFκBp65* nuclear factor kappa-light-chain enhancer of activated B cells p65 subunit, *PE* phosphatidyl ethanolamine, *PS* phosphatidyl serine, *PC* phosphatidyl choline, *MAP2K1* dual specificity mitogen-activated protein kinase kinase 1, *PGE* prostaglandin E2, *EP* E-prostanoid, *mPGES* microsomal prostaglandin synthase E, *cPLA* cytosolic phospholipase A, *COX* cyclo-oxygenase, *ICAM* cell adhesion molecule, *TIMP* tissue inhibitors of metalloproteinases, *sST* suppressor of T-cell receptor signaling, *TBARS* thiobarbituric acid reactive substance, *HT* hydroxytryptamine, *GABA* gamma-aminobutyric acid, *IFI* interferon-γ-inducible protein, *HSP* heat shock proteins, *CD* cluster of differentiation, *L* ligand, *Flt3* Fms-related tyrosine kinase, *VIP* vasoactive intestinal peptide, *NT* neurotrophin, *NO* nitric oxide, *MMP* matrix metalloproteinase, *ASD* autism spectrum disorder, *HC* healthy controls, *ASD-C* childhood autism, *ASD-AS* Asperger syndrome, *LPS* lipopolysaccharides, *GI* gastrointestinal symptoms, *RTT* Rett syndrome, *PHA* phytohemagglutinin, *I* inflammatory subtype (defined as fluctuating behavioral symptoms following immune insults), *NFA* non-IgE-mediated food allergy, *SPAD* specific polysaccharide antibody deficiency, *TLR* Toll-like receptors, *SIB* siblings, *DD* developmental delay, *NHF* non-allergic food hypersensitivity, *DPI* dietary protein intolerance, *ABC* Aberrant Behavior Checklist, *BMI* body mass index, *IQ* intelligence quotient, *ADI-R* Autism Diagnostic Interview–Revised, *MSEL* Mullen Scales of Early Learning, *VABS* Vineland Adaptive Behavior Scale, *BDE-47* 2,2′,4,4′-tetrabromodiphenyl ether, *ADOS* Autism Diagnostic Observation Schedule

^A^Abstract

Monocytes are a part of an innate immune system that differentiate into macrophages and migrate into the surrounding tissue where they present antigens to lymphocytes [186]. State-of-the-art research has shown that current understanding of monocyte and macrophage biology is insufficient and should undergo revision [187]. Monocytes in an inflammatory milieu have been known to secrete proinflammatory mediators such as IL-1β, IL-8, or TNF-α, and their prolonged activation has been found to be relevant in the course of rheumatoid arthritis [188], Alzheimer’s [189], and Parkinson’s disease [190]. Changes in monocyte function observed in ASD may not be causative, as studies suggest that monocytes are in an activated state and their proinflammatory activity could add on to existing immune imbalances, exacerbating behavioral symptoms.

## Microglia

Microglia, resident innate immune cells of the brain, are specialized tissue macrophages of the CNS that monitor brain homeostasis [191]. They are known to play an important role in the pathogenesis of neuropsychiatric disorders including ASD [192–195]. During brain development, microglia take part in synaptic and neuronal development and regulation of stem cell proliferation [196]. In autistic children, microglia are located closer to neurons and activated in several regions of brain, especially in cerebellum [196, 197]. Morgan et al.’s study [198] of dorsolateral prefrontal cortex microglia revealed marked activation in 5 of 13 studied individuals with ASD (especially in children under age of 6) and marginal activation in 4. Moreover, microglial volume and cell density were increased in ASD subjects, which is in accordance with other studies [199]. Activation of microglia may be linked to abnormal brain connectivity reported in children with ASD [200, 201]. Studies in mouse models of obsessive compulsive disorder and trichotillomania with microglial abnormalities revealed that after transplantation of bone marrow-derived stem cells, key symptoms of the disease were ameliorated [202]. This result suggests that modulation of immune system can lead to behavioral improvements.

Microglial abnormality is a promising area that should be more intensively researched. Resident immune cells have been found to play a role in white matter abnormalities in the brain of schizophrenia patients [204, 205]. The interplay between the peripheral immune system and microglia [205–207] as well as abnormal white matter connectivity found in ASD [208, 209] makes microglia an interesting target for further studies in this field. Finding explicit connections between microglial activation and peripheral immune abnormalities in ASD could uncover new possibilities for targeted interventions.

## Cytokines and chemokines in brain tissue and cerebrospinal fluid

A tremendous insight into ASD pathogenesis was achieved through cytokine studies on autistic brains. Frozen tissue lysates of front cerebral cortex from eight ASD patients and matched individuals were compared for concentrations of several cytokines. The subjects were aged 4–37 years (mean age of 12) and included five males and three females with moderate to severe ASD. The control group was age- and sex-matched and included individuals that had been diagnosed with asthma, heart disease, or other injuries. TNF-α, IL-6, granulocyte colony-stimulating factor (G-CSF), IFN-γ, and IL-8 were increased in the brain of ASD patients. There were no differences in IL-1β, IL-2, IL-4, IL-5, or IL-10. The study revealed that proinflammatory and Th1 cytokines as well as Th1/Th2 ratio (measured as IFN-γ/IL-10 ratio) and IL-8 were up-regulated in comparison with control group [210]. Vargas et al. [211] studied cytokine profiles in both brain and CSF. The brain tissue was sampled from middle frontal gyrus, anterior cingulate gyrus, and cerebellar hemisphere. The study group was composed of 15 patients (3 females) aged 5–44; most had been diagnosed with mental retardation (12/15) and some with epilepsy (6/15). The control group consisted of 12 individuals, including 3 females, aged 5–46, with no neurological disorders. The causes of death in both groups were non-neurological. The expression of 79 proteins, including cytokines, chemokines, and growth and differentiation factors, was measured on seven autistic brains and controls by protein array studies and confirmed with ELISA array for the most significant proteins. The most prominent changes were captured in anterior cingulate gyrus; however, several proteins turned out to be up-regulated in all studied regions (MCP-1, TARC,TGF-β1) and one in CSF as well (MCP-1). Proteins up-regulated in CSF included IL-6, INF-γ, and IL-10. Interestingly, immunocytochemical staining was carried out to detect structures responsible for increased cytokine concentration and revealed that astrocytes localized in the cerebellum and cortical and subcortical white matter regions were the main source of MCP-1 and IL-6. Moreover, Vargas et al. [211] found that microglia and astroglia activation was most prominent in the cerebellum. However, interpretation of this study should be careful due to the co-existing pathologies of epilepsy and mental retardation. Elevated concentration of IL-6 was also found by another study that examined cerebellar brain tissue derived from six ASD individuals compared to six control subjects [212]. Zimmerman et al.’s study [213] aimed to examine CSF. They obtained CSF and serum from 12 patients with moderate to severe ASD, including 2 females, aged 2.7–10 years, most with regression, and some with epilepsy or abnormal EEG results. The study revealed an increased concentration of biopterin and a decreased concentration of quinolinic acid and neopterin (indicators for immune activation) in comparison with control group; however, those changes could be due to the fact that control group was composed of patients with central nervous system diseases. Cytokine profile showed a higher serum soluble TNF-α receptor II concentration in children with ASD compared with siblings, normal children, and patients with central nervous system diseases. No other abnormalities were detected. No correlation between serum and CSF concentration was observed and presence of regression did not seem to influence results in any manner. The authors concluded that decreased quinolinic acid and neopterin along with increased biopterin in CSF might be a result of metabolic pathway dysmaturation in the absence of co-existing infection or due to expression localized to microglia. Another study [214] that examined pterin compounds in CSF found 7,8-dihydroneopterin and 6R-5,6,7,8-tetrahydrobiopterin to be significantly down-regulated in comparison with controls. Several other investigators have raised the issue of elevated pterins in both serum and urine as a sign of cellular immunity activation, stimulated by IFN-γ and TNF-α with conflicting results [161, 215–218]. The earliest study on 16 ASD children and 12 typically developing controls found decreased plasma and urinary levels of neopterin and monapterin accompanied by normal tetrahydrobiopterin level [218]. In contrast, two other studies on urinary pterins revealed that both neopterin and biopterin were elevated in ASD individuals in comparison to healthy controls [216, 217]. High plasma neopterin in ASD individuals was also confirmed in two other studies [161, 215]. Several attempts to treat ASD with tetrahydrobiopterin and sapropterin have been carried out demonstrating potentially positive effects, including three clinical trials [219–224]. However, molecular studies on ASD individuals revealed only one marginal association with a gene responsible for tetrahydrobiopterin synthesis [225]. Currently, there are no open clinical trials in this area.

An elevation of NF-κB in neurons and microglia was found to be significant in orbitofrontal cortex of ASD individuals [226]. Chez et al. [227] evaluated the concentration of TNF-α simultaneously in both serum and CSF of 10 male patients aged 2.5–9.7 with regressive ASD. The study’s results must be interpreted cautiously since 7 out of 10 patients were on medications, including valproic acid and risperidone, which are thought to have anti-inflammatory and potentially immunomodulatory properties [228–231]. Four patients had received treatment for autoimmunity in the past, but no details concerning timeframe of treatment were provided. The ratio of TNF-α in CSF and serum ranged between 1.7 and 275, with an average value of 41.6, and the concentration of TNF-α in CSF and the CSF/serum ratio were higher in patients who did not undergo immunomodulatory therapy. The authors hypothesized they may have observed a unique CNS response, as no apparent correlation exists between CSF and serum and the CSF/serum ratio described in other diseases (HIV, ischemic stroke, traumatic brain injury, multiple sclerosis, systemic lupus erythematosus, frontotemporal dementia) is close to 1:1. A similar hypothesis concerning lack of association between different protein concentration in CSF and serum was confirmed by Pardo et al. [232]. The results of studies conducted on CSF and brain tissue are summarized in Table 4.

**Table 4.** Concentration of cytokines, chemokines, and growth factors in brain tissue and cerebrospinal fluid of ASD patients

Number	Study	Study group age (years)	Groups	Females (%)	Death causes	Study material	Cytokines	Chemokines	Growth factors	Main results	Excluded correlations
1	Pardo et al. 2017 [232]	R: 2–8	ASD (*n* = 104, 67 for CSF analysis), HC (*n* = 54)	ASD (17), HC (24)	NA	CSF, serum	IL-1α, IL-1RA, IL-1β, IL-2, sIL-2RA, IL-3, IL-4, IL-5, IL-6, IL-7, IL-9, IL-10, IL-12p40, IL-12p70, IL-13, IL-15, IL-17, IFN-α2, IFN-γ, TNF-α, TNF-β, TGF-α	CCL2 (MCP-1), CCL3 (MIP-1α), CCL4 (MIP-1β), CCL7 (MCP-3), CCL11 (eotaxin), CCL22 (MDC), CXCL1 (GRO-α), CXCL8 (IL-8), CXCL10 (IP-10), CX3CL1 (fractalkine)	EGF, G-CSF, GM-CSF, VEGF, FGF-2, FLT-3L, sCD40L	ASD vs. TD: ↑EGF, sCD40L (serum), high within-subject variation of studied parameters	No association between concentrations of studied parameters in blood and CSF
2	Wei et al. 2010 [212]	M: 8.3, SD: 3.8	ASD (*n* = 6), HC (*n* = 6)	ASD (33), HC (33)	ASD: drowning (*n* = 4), multiple injuries (*n* = 1), smoke inhalation (*n* = 1); HC: drowning (*n* = 2), multiple injuries (*n* = 1), cardiac arrhythmia (*n* = 1), asthma (*n* = 1), lymphocytic myocarditis (*n* = 1)	Brain tissue (cerebellum)	IL-6	x	x	ASD vs. TD: ↑IL-6	x
3	Li et al. 2009 [210]	M: 12.8, R: 4–37	ASD (*n* = 8), HC (*n* = 8)	ASD (37), HC (37)	No data	Brain tissue (front cerebral cortex)	IL-1β, IL-2, IL-4, IL-5, IL-6, IL-10, IFN-γ, TNF-α	CXCL8 (IL-8)	GM-CSF	ASD vs. TD: ↑IL-6, IFN-γ, TNF-α, IL-8, GM-CSF, Th1/Th2 ratio	No differences in IL-1β, IL-4, IL-5, IL-10 concentration
4	Chez et al. 2007 [227]	M: 5.4, SD: 2.1, R: 2.5–9.5	Regressive ASD (*n* = 10), LGS (*n* = 1)	ASD (0), LGS (0)	NA	CSF, serum	TNF-α	x	x	CSF vs. serum: ↑TNF-α; ASD + medications: ↑TNF-α, ASD vs. Lennox–Gastaut: ↑TNF-α	No abnormalities in CSF including protein, glucose, glutamate levels, myelin basic protein, oligoclonal bands
5	Zimmerman et al. 2005 [213]	ASD1—M: 6.1, R: 1.4–10; ASD2—M: 7.8, R: 2.8–43	ASD1 (*n* = 12), ASD2 (*n* = 35, including 1 with ASD-AS, 1 with PDD-NOS, 1 with high functioning ASD), HC1 (*n* = 15), HC2 (*n* = 12), HC3 (*n* = 2), SIB (*n* = 10), ND (*n* = 11)	ASD1 (17), ASD2 (8), HC1 (60), HC2 (50), HC3 (50), SIB (50), ND (9)	NA	CSF (ASD1, HC1, HC2), serum (ASD1, ASD2, HC3, ND, SIB)	IL-RA, IL-6, IL-1β, IL-2, IFN-γ, TGF-β, sTNFRI, sTNFRII	x	x	ASD vs. TD: ↑quinolinic acid, neopterin ↓biopterin; ASD vs. ND: ↑biopterin ↓quinolinic acid, neopterin; ASD vs. ND and SIB: ↑sTNFRII	No abnormalities in CSF including protein, glucose, oligoclonal bands. IL-1β, IL-2, IFN-γ, TGF-β not detected in CSF
6	Vargas et al. 2005 [211]	Brain tissue—M: 12, R: 5–44; CSF—M: 5.5, R: 4–10	Brain tissue: ASD (*n* = 15), HC (*n* = 12); CSF: ASD (*n* = 6), HC (*n* = 9)	Brain tissue: ASD (20), HC (25); CSF: ASD (33), HC (67)	ASD: drowning (*n* = 5), trauma sudden death (*n* = 3), respiratory failure (*n* = 1), hyperthermia (*n* = 1), trauma (*n* = 1), aspiration (*n* = 1), acute myocardial infarction (*n* = 1), unknown (*n* = 1); HC: drowning (*n* = 1), allograft rejection (*n* = 1), cardiac arrhythmia (*n* = 1), accidental (*n* = 1), asthma (*n* = 1), gunshot (*n* = 1), trauma (*n* = 2), hanging (*n* = 1), asphyxia (*n* = 1), heart disease (*n* = 1), sudden death (*n* = 1)	Brain tissue (cerebellum, midfrontal, and cingulate gyrus), CSF	IL-1α, IL-1β, IL-2, IL-3, IL-4, IL-5, IL-6, IL-7, IL-10, IL-12, IL-13, IL-15, IL-16, IFN-γ, TNF-α, TNF-β, TGF-β1, TGF-β2, TGF-β3	CCL1 (I-309), CCL2 (MCP-1), CCL3 (MIP-1α), CCL4 (MIP-1β), CCL5 (RANTES), CCL7 (MCP-3), CCL8 (MCP-2), CCL9 (MIP-1γ), CCL11 (eotaxin), CCL22 (MDC), CCL24 (eotaxin-2), CCL26 (eotaxin-3), GRO, CXCL1 (GRO-α), CXCL8 (IL-8), CXCL9 (MIG), CXCL10 (IP-10), CX3CL1 (fractalkine), ENA-78, MIP-1gamma, SDF-1, CCL17 (TARC), BLC, GCP-2, MCP-4, MIF, MIP-3alpha, NAP-2, CkBeta8-1	G-CSF, M-CSF, SCF, EGF, IGF-I, Ang, OSM, Tpo, VEGF, PDGF-B, Leptin, BDNF, FGF-4, FGF-6, FGF-7, FGF-9, Flt-3 ligand, GDNF, HGF, GFBP-1, IGFBP-2, IGFBP-3, IGFBP-4, LIF, LIGHT, NT-3, NT-4, Osteoprotegerin, PARC, PIGF, TIMP-1, TIMP-2	ASD vs. TD: ↑IL-6, IL-10, CCL2 (MCP-1), CCL7 (MCP-3), CCL8 (MCP-2), TGF-β1, IGFBP1, CCL11 (eotaxin), eotaxin-2, TARC, MDC, Ckβ8.1, MIG, BLC, IGF-1, leptin, Flt3-lig, IGFBP1, osteoprotegerin, microglia and astroglia activation	No evidence of adaptive immune reactions

*R* range, *M* mean, *SD* standard deviation, *ASD* autism spectrum disorder, *CSF* cerebrospinal fluid, *HC* healthy controls, *LGS* Lennox–Gastaut Syndrome, *SIB* siblings, *ND* neurological disorders, *IL* interleukin, *IFN* interferon, *TNF* tumor necrosis factor, *TGF* transforming growth factor, *sR* soluble receptor, *CCL* C-C motif chemokine ligand, *MCP* monocyte chemoattractant protein, *MIP* macrophage inflammatory protein, *MDC* macrophage-derived chemokine, *CXCL* C-X-C motif chemokine ligand, *GRO* growth-regulated oncogene, *IP-10* IFN-γ-inducible protein 10, *MIG* monokine induced by interferon-gamma, *ENA-78* epithelial cell-derived neutrophil activating peptide-78, *SDF* stromal cell-derived factor, *TARC* thymus and activation-regulated chemokine, *EGF* epidermal growth factor, *G-CSF* granulocyte colony-stimulating factor, *GM-CSF* granulocyte-macrophage colony-stimulating factor, *VEGF* vascular endothelial growth factor, *BDNF* brain-derived neurotrophic factor

It is worth noting that though all of the described studies detected abnormalities in CSF or brain tissue of ASD patients, high variability between subjects, small samples, and lack of correlation between clinical symptoms and laboratory results hinder interpretation. Only three studies [213, 227, 232] focused on both CSF and peripheral blood and did not confirm any correlation between the same proteins in those two samples. It would be highly beneficial to conduct larger studies, simultaneously evaluating concentrations of proteins in both CSF and peripheral blood. It is understandable that lumbar puncture for scientific purposes might be unacceptable to some patients’ parents as it is connected with high levels of child stress and/or administration of premedication or anesthetics. However, ASD children often undergo MRI scans under general anesthesia for diagnostic purposes and CSF sampling could be done at that time and preserved for further studies. Understanding differences and interactions between the periphery and CNS is crucial for determining novel therapeutic strategies.

## Peripheral blood cytokines, chemokines, and growth factors

The first studies on plasma cytokine levels in individuals with ASD showed increased levels of IFN-γ, IL-2, and IL-12 and thus concluded that ASD may be linked to pathological stimulation of Th1 cells. No difference between the study and control group was found for IFN-α, IL-1, IL-6, TNF-α, or soluble intercellular adhesion molecule-1 [149, 160]. An increase in the Th1 inflammatory response was also found by Croonenberghs et al. [182] who studied production of IL-6, IL-10, interleukin-1 receptor antagonist (IL-1RA), IFN-γ, and TNF-α in whole blood and IL-6, interleukin-2 receptor (IL-2R), and IL-1RA in serum of ASD individuals. The study revealed increased levels of IFN-γ and IL-1RA in the supernatant of ASD group whole blood cultures. A relationship between Th1 and Th2 cytokines was also studied by Gupta et al. [88]. They found an imbalance between Th1 and Th2 cytokines with increased IL-4^+^CD4^+^ T cells and IL-4^+^CD8^+^ T cells and decreased proportions of IFN-γ^+^CD4^+^ T cells, IL-2^+^CD4^+^ T cells, and IFN-γ^+^CD8^+^ and IL-2^+^CD8^+^ T cells in children with autism. Molloy et al. [148] compared production of several cytokines (IL-2, IL-4, IL-5, IL-10, IL-13, IFN-γ) in PB-MNC of ASD children and healthy controls and found a predominance of Th2 response with an imbalance in Th1/Th2 cytokine subsets in the ASD children MNC. Production of TNF-α, IL-1β, and IL-6 by PB-MNC was significantly increased with and without stimuli (PHA, tetanus, IL-12p70, IL-18) [159] in the ASD group. The same investigators measured cytokine production against common dietary proteins and found increased proinflammatory cytokine responses (IFN-γ and TNF-α) that might predispose ASD individuals to GI inflammation and worsen disease behavioral symptoms [157]. In another study, both children with ASD and non-allergic food hypersensitivity had elevated cytokine production after stimulation with common dietary proteins and similar cytokine profiles. Both groups had high TNF-α and IL-12 concentrations and individuals with non-allergic food hypersensitivity also had elevated IFN-γ and IL-10 levels [183] (Table 3).

Suzuki et al. [141] measured concentration of multiple proteins in plasma of high-functioning male children with ASD aged 7–15. The study included 21 children with ASD disorder and 7 with pervasive developmental disorder–not otherwise specified (PDD-NOS). The investigators found that IL-1β, IL-1RA, IL-5, IL-8, IL-12p70, IL-13, IL-17, and growth-regulated oncogene-α were significantly elevated (1.5–2.5-fold) in comparison to matched controls; however, no correlation between clinical profile and laboratory results was observed. An interesting contribution was made by Han et al. [164] who found distinct chemokine and cytokine profiles between ASD individuals and ASD children with comorbid diagnosis of attention deficit hyperactivity disorder (ADHD). The study group consisted of children aged 6–17 years, 9 with ASD and ADHD, 13 with ASD only, and 13 typically developing controls matched by age, gender, and IQ. Investigators compared concentrations of several chemokines and two cytokines (TGFβ1 and macrophage migration inhibitory factor—MIF) of which MCP-1 and Th2-related RANTES were significantly higher and Th1-related C-X-C motif chemokine ligand (CXCL) 9 was lower in all children with ASD, in comparison to healthy controls. Moreover, an increased MIF and decreased CXCL10 concentration was found to correlate with lower executive functioning scores, while CXCL9 was inversely correlated with short-term memory function. Increased concentration of RANTES and decreased CXCL9 were associated with poor behavioral scores (social domain, repetitive behavior, and hyperactivity). Children with co-morbid ADHD had higher MIF and lower IL-8 concentration than ASD-only children. Individuals with ASD and ADHD were different from typically developing controls in concentration of MIF (higher) and in IL-8 and CXCL9 (lower).

Frequently, ASD individuals were reported to have a higher concentration of proinflammatory or lower concentration of anti-inflammatory cytokines [93, 135, 137, 141–143, 145, 146, 149, 151, 152, 155, 157–160, 163, 169–171, 182, 183] in comparison to healthy controls or other developmental delays, although some results are contradictory [88, 91, 139, 144, 147, 148, 153, 154, 156, 166, 172, 173, 177]. Interestingly, Tsilioni et al. [170] distinguished two autistic groups—with initially low or high IL-6 and TNF levels, whereas another study divided subjects according to response to LPS stimulation and found high levels of IL-1β and IL-6 in LPS responders [150]. One recent study focused on several soluble factors that have not previously been studied in ASD. Investigators researched IL-21, IL-22, IL-27, and cytotoxic T-lymphocyte-associated molecule-4 as indicators of pro- and anti-inflammatory balance and revealed dysregulation of immune milieu [162]. Other differences between ASD subjects and healthy individuals included high MCP-1, RANTES, eotaxin [174], TARC, MDC [89], BDNF, and platelet-derived growth factor (PDGF) concentrations [140, 152], low epidermal growth factor (EGF) [139, 175], and altered IL-23 [96, 152–154, 156, 178] and IL-8 [141, 142, 144, 145, 147, 169]. Intriguingly, a few studies included normally developing siblings as one of the control groups and found that their biomarker profile was distinct from other normally developing children [91, 179, 183].

Several published studies did not confirm differences between ASD and healthy individuals in baseline or stimulated levels of cytokines, chemokines, or growth factors [94, 136, 142, 147, 153, 168, 170, 181].

Approximately half of the studies tried to correlate behavioral profile with laboratory abnormalities (Table 3). One of them did not find any correlation with clinical measures [168]; however, it evaluated only two cytokines (IL-1β, IL-33), scored children with the Social Responsiveness Scale (SRS) alone, and included those also on psychotropic medication. However, there were no differences found between patients on and off medication.

A link between ASD severity and cytokine or chemokine abnormalities was sought for extensively. Increased concentration of IL-1β [150–152], IL-6 [135, 150, 171], IL-12p70 [135], IL-17A [93], TNF-α [135], MIF [179], MDC, and TARC [89] positively correlated with more severe behavioral symptoms. Only one out of seven studies on this topic failed to confirm a connection between peripheral blood cytokines (TNF-α, IL-1, IL-6) and disease severity [180].

Several investigators identified a relationship between social sphere and concentration of several factors, out of which IL-6 was repeatedly found to be relevant. IL-6 correlated positively with social impairments both at baseline level [150, 167] and after stimulation [155]. Up-regulation of IL-1β [150, 155], IL-10 [150], MCP-1 [150, 219], MIP-1β [138], MIP-1δ [138], MIF [180], and endotoxins [177] and down-regulation of IL-23 [96], TGF-β1 [177], TNF-α [166], and GM-CSF [138] were also described in relation to social dysfunction.

In opposition to the above-described results, Han et al. [164] found no association of social domain with TGF-β1 and MIF. Correlation with social impairment was also negative in two other experiments that focused on IL-10, IL-12 [73], PDGF, and vascular endothelial growth factor (VEGF) [185]. Poorer performance on Vineland Adaptive Behavior Scale was connected with low GM-CSF, IL-1β, IL-2, IL-6, and MCP-1, with high MIP-1δ [138], and no association was found with IL-17, IL-23, and TGF-β1 [96, 177].

Deficits in communication and language were frequently found to be associated with an impaired protein profile, in particular higher concentration of IL-4, IL-8, IL12p40, IFN-γ [144, 145], MIP-1α, and RANTES [174] and lower concentrations of IL-5 and IL-10 [144]. Concentration of PDGF and VEGF [185] and mRNA levels of IL-1β, IL-6, and TNF-α [166] were found to be independent of communication skills. Poor verbal contact was linked with up-regulation of IL-1β and IL-6 after stimulation [155] as well as high concentration of IL-10, GM-CSF, and M-CSF [138].

Stereotypic behavior seemed to correlate with down-regulation of TGF-β1 [177] and GM-CSF [144] as well as up-regulation of IL-1β, IL-6, IL-8, IL12p40, TNF-α, and IFN-γ [144, 145]. Restricted patterns of behavior and interests were more pronounced in patients with high concentration of MCP-1, RANTES, eotaxin [174], and PDGF-BB [185].

Surprisingly, exacerbations of both hyperactivity and lethargy were found to be linked to low levels of anti-inflammatory cytokines (IL-10, TGF-β), high levels of proinflammatory cytokines (IL-1β, IL-6) [145, 151], several chemokines (IL-8, RANTES, eotaxin), and high response to PHA stimulation [144, 174]. Hyperactivity was also associated with low levels of CXCL5 [164] and IL-13 and high levels of IL-12p40 [144]. Irritability was also associated with a similar balance of pro- and anti-inflammatory cytokines [151] apart from a low level of TNF-α [167] or TGF-β1 [178].

Most of the studies did not reveal any association between IQ and soluble molecules profile [145, 176, 185] apart from low concentration of IL-6 in Ferguson et al. [167] study and Napolioni et al. research [138] in which a wide range of cytokines inversely correlated with IQ. Impaired short-term memory was associated with high CXCL9 [164].

An interesting observation about sleep disturbances and aggressive behavior was made by Careaga et al. who found up-regulation of IL-1β, IL-6, IL-10, and MCP-1 in children whose PBMNC responded well to LPS stimulation [150]. Interestingly, Th-1 skewed response was associated with more severe developmental impairment.

Other studied areas included fine motor skills (down-regulated IL-5, up-regulated MCP-1, RANTES, and eotaxin), visual reception (up-regulated IL-8, MCP-1, RANTES, and eotaxin) [144, 176], executive functioning (high MIF, low CXCL10) [164], daily living abilities (high MCP-1, eotaxin), and adaptive and cognitive functions (high MCP-1, RANTES, and eotaxin, low TGF-β1 and GM-CSF, and no association with IL-10 or IL-12) [73, 138, 144, 174, 177].

When analyzing various developmental patterns of ASD patients it would seem that children who lost abilities would present with different biological conditions. A few studies that researched a wide cytokine profile negated its connection with regression [144, 163]. However, Ashwood et al. [145] showed that children who regressed expressed higher levels of IL-1β in comparison to other ASD individuals and higher levels of IL-1β, IL-6, IL-8, and GM-CSF in comparison to healthy controls. IL-1β was also found significantly higher by Napolioni et al. [138] along with IL-5 and IL-17 in children with regressive ASD. A single study reported a connection between higher TNF-α concentration and occurrence of regression [167].

GI issues are frequently reported among autistic individuals [233]. PB-MNC from ASD individuals with GI problems were found to have altered concentration of several cytokines; however, results are inconsistent [137, 138, 154, 158, 167, 184]. ASD children with GI symptoms were also assessed for intracellular cytokines in CD3^+^ lymphocytes in both peripheral blood and mucosa. Peripheral blood levels of TNF-α were similar to typically developing children with Crohn disease and were increased in comparison to healthy controls. Similar observations were made for IFN-γ both in peripheral blood and terminal ileum lymphocytes. IL-10 was down-regulated in ASD children with GI symptoms in comparison to both healthy controls and children with Crohn disease in both peripheral blood and terminal ileum mucosa. The observed differences were maintained following stimulation [234]. Lymphocytic colitis in ASD children, especially those with GI symptoms and regression, was reported in several papers. A study on immunological aberrations in gut mucosa, based on duodenal, ileal, and colonic biopsies, revealed up-regulation of CD3^+^CD8^+^ intraepithelial lymphocytes and CD3^+^ lamina propria lymphocytes with proinflammatory cytokine profile [234, 235]. Functional GI disorders in children with ASD were associated with elevation of several proinflammatory cytokines in rectal biopsy mucosa [236]. ASD children with GI complaints were found to have comparable levels of CD3^+^ lymphocyte intracellular cytokines [237] and even higher intraepithelial cell number and CD8^+^ density than children with Crohn disease [238].

Another study revealed epithelial IgG and complement deposition in almost all children with regressive ASD [239]. However, not all investigators were successful in determining abnormalities of intestinal biopsy specimens in ASD individuals [240].

An unusual approach was proposed in a double-blind, placebo-controlled trial with camel milk, assessing whether 2-week administration of raw or boiled camel milk, instead of cow milk, would help to reduce serum levels of TARC and lessen autistic traits. Camel milk is said to have unique properties such as low molecular weight immunoglobulins. Investigators hypothesized that camel milk could down-regulate synthesis and secretion of TARC leading to reduction of inflammatory processes. Best behavioral outcomes were observed with raw camel milk; however, the use of both forms of camel milk led to significant reduction of TARC levels [241].

Multiple studies have confirmed cytokine, chemokine, and growth factor abnormalities in ASD. For details concerning demographic details and medical history of analyzed patients, please refer to Table 2; for summarized results, please see Table 3. The main results concern proinflammatory cytokines. IL-1 was found to be found up-regulated frequently and its high concentration was connected with regression [138, 145], ASD severity [152], deficits in social sphere [150, 155], impaired adaptive skills [138] and development [150], as well as hyperactivity, lethargy, and irritability [151]. IL-6 was strongly associated with ASD severity [135, 171] and deficits in social sphere [150, 155, 167]. Its up-regulation, analogous to IL-1, was found to be significantly correlated with hyperactivity, lethargy, and irritability [151]. Interestingly, higher IQ was connected with lower IL-6 level [138, 167]. For detailed summary, please refer to Table 5.

**Table 5:** Association between autistic traits, co-occurring symptoms, and immune-specific molecules in peripheral blood of ASD children

Type of analyte	Analytes	ASD severity	Regression	Impaired development	Deficits in social sphere	Impaired communication	Repetitive behaviors and interests, stereotypies	Impaired cognitive sphere	Impaired adaptive skills	Impaired imaginative skills	Aggression	Hyperactivity	Irritability	Lethargy	Anxiety	Higher IQ	Short-term memory	Attention deficits	Sleep disturbances	GI symptoms	Epilepsy or EEG abnormalities	Head circumference
Cytokines	1. IL-1^a^ [91, 135–147, 149–156, 159, 163, 166, 168, 169, 174, 179, 181, 183, 184]	↑ (1/4) [152]	↑ (2/4)[138, 145]	↑ (1/1)[150]	↑ (2/5)[150, 155]	↑ (1/4)[155]	↑ (1/2)[145]	x	↑ (1/3)[138]	NA	↑ (1/1) [150]	1. ↑ (1/2)[151]	↑ (1/2)[151]	↑ (1/2)[151]	NA	↓ (1/2)[138]	NA	NA	↑ (1/1)[150]	↑ (1/2)[138]	x	NA
	2. IL-2 [88, 91, 135, 136, 138–141, 143–149, 156, 162]	x	x	NA	x	x	x	x	↑ (1/1)[138]	NA	NA	2. x	x	x	NA	x	NA	NA	NA	↑ (1/2)[138]	x	x
	3. IL-4 [88, 94, 135, 136, 138–141, 143–148, 155, 159, 163, 183]	x	x	NA	x	↑ (1/4)[145]	x	x	x	NA	x	3. x	x	x	NA	x	NA	x	NA	x	x	x
	4. IL-5 [135–141, 144–148, 154, 155, 157, 159, 183]	x	↑ (1/3)[138]	NA	x	x	x	↓(1/2)[144]	x	NA	x	4. x	x	x	NA	x	NA	x	NA	x	x	x
	5. IL-6 [88, 91, 135, 136, 138–147, 150–156, 158–160, 163, 165–167, 170–172, 174, 180, 182, 183]	↑ (2/4) [135, 171]	↑ (1/5)[145]	↑ [150]	↑ (3/6)[150, 155, 167]	↑ (2/5)[140, 171]	↑ (1/2)[145]	x	↑ (1/3)[138]	NA	↑ (1/3)[150]	5. ↑ (1/3)[150]	↑ (1/2)[151]	↑ (1/3)[151]	x	↓ (2/4)[138, 167]	NA	x	↑ (1/1)[150]	↑ (1/3)[138]	↑ (1/1)[135]	x
	6. IL-6sR [135, 138]	x	x	NA	↑ (1/1)[138]	x	NA	NA	x	NA	NA	6. NA	NA	NA	NA	x	NA	NA	NA	x	x	x
	7. IL-7 [135, 136, 138–141]	x	x	NA	NA	x	NA	NA	x	NA	NA	7. NA	NA	NA	NA	↓ (1/1) [138]	NA	NA	NA	x	x	x
	8. IL-10 [73, 88, 91, 135, 136, 138–148, 150, 151, 153–159, 163, 174, 182, 184]	x	x	↑ [150]	↑ (1/5)[150]	↑ (1/4)[138]	x	x	x	NA	↑ (1/1)[150]	8. ↓ (1/3)[151]	↓ (1/1)[151]	↓ (1/3) [139]	NA	x	NA	x	↑ (1/1)[150]	x	x	x
	9. IL-11 [138, 140]	NA	x	NA	x	x	NA	NA	x	NA	NA	9. NA	NA	NA	NA	↓ (1/1)[138]	NA	NA	NA	x	NA	x
	10. IL-12 [73, 135, 136, 139–142, 144, 145, 150–155]	↑p70, ↓p40 (1/3)[135]	x	x	x	↑ (1/4)[144]	↑ (1/3)[145]	x	x	NA	x	10. ↑ (1/3)[144]	x	↑ (1/2)[145]	NA	↓ p70 [138]	NA	x	x	x	↑ (1/1)[135]	x
	11. IL-13 [91, 94, 135, 136, 138–141, 144, 145, 147, 148, 150, 152, 156–160]	x	x	x	x	x	x	x	x	NA	x	11. ↓ (1/1) [80]	x	x	NA	↓ (1/1)[138]	NA	NA	x	x	x	x
	12. IL-16 [135, 138, 140, 141]	x	x	NA	x	x	NA	NA	x	NA	NA	12. NA	NA	NA	NA	↓ (1/1)[138]	NA	NA	NA	x	x	x
	13. IL-17 [93, 94, 96, 135–141, 150, 151, 154, 163, 178]	↑ (1/2)[93]	↑ (1/1)[138]	x	x	x	NA	x	x	NA	x	13. x	x	x	NA	↓ (1/1)[138]	NA	NA	x	x	x	x
	14. IL-23 [96, 140, 151–154, 156, 178]	x	NA	NA	↓ (1/1)[96]	NA	NA	x	x	NA	NA	14. x	x	x	NA	NA	NA	NA	NA	NA	NA	NA
	15. IFN-γ [135, 136, 138–140, 142–146, 150, 163, 172, 173]	x	x	x	x	↑ (1/1)[144]	↑ (1/2)[144]	x	x	NA	x	15. x	x	x	NA	x	NA	x	x	x	x	x
	16. TNF-α [135, 137, 138, 144, 145, 150–152, 155, 163, 166, 167, 180]	↑ (1/2)[135]	x	x	↓ (1/4)[166]	x	↑ (1/2)[144]	x	x	NA	x	16. x	↓ (1/2)[167]	x	↑ (1/1)[167]	x	NA	x	x	↑ (1/1)[167]	x	x
	17. TNF-sRII [135, 138]	x	x	NA	x	x	NA	NA	x	NA	NA	17. x	x	x	NA	↓ (1/1)[138]	NA	NA	NA	x	NA	x
	18. TGF-β [139, 151, 153, 159, 164, 173, 177]	NA	x	NA	↓ (1/2)[177]	x	↓ (1/2)[177]	x	x	NA	NA	18. ↓ (1/2)[177]	↓ (1/2)[177]	↓ (1/2)[177]	NA	NA	x	x	NA	NA	NA	NA
	19. MIF [140, 164, 179]	↑ (1/1)[179]	NA	NA	↑ (1/2)[179]	x	x	↑ (1/1)[164]	NA	↑ (1/1)[179]	NA	19. x	NA	NA	NA	NA	x	x	NA	NA	NA	NA
Chemokines	1. CCL2 (MCP-1) [135, 136, 138–141, 147, 150, 155, 164, 175]	x	x	↑ (1/1)[150]	↑ (1/2)[174]	x	↑ (1/1)[174]	↑ (1/4)[174]	↑ [138, 174]	NA	↑ (1/1)[150]	1. x	x	x	NA	x	x	x	↑ (1/1)[150]	x	x	x
	2. CCL4 (MIP-1β) [135, 136, 138–141, 147, 175]	x	x	NA	↑ (1/1)[138]	x	x	x	x	NA	NA	2. x	x	x	NA	x	NA	NA	NA	x	x	x
	3. CCL5 (RANTES) [135, 136, 138–141, 147, 164, 174]	x	x	NA	x	↑ (1/2)[174]	↑ (1/1)[174]	↑ (1/1)[174]	↑ (1/2)[174]	NA	NA	3. ↑ (1/1)[174]	x	↑ (1/1)[174]	NA	x	x	↓ (1/1)[164]	NA	x	x	x
	4. CCL11 (eotaxin) [135, 136, 138–141, 147, 175]	x	x	NA	x	x	↑ (1/1)[174]	↑ (1/1)[174]	↑ (1/2)[174]	NA	NA	4. ↑ (1/1)[138]	x	↑ (1/1)[174]	NA	x	NA	NA	NA	x	x	x
	5. CCL15 (MIP-1δ) [138]	x	x	x	↑ (1/1)[138]	x	x	x	↓ (1/1)[138]	NA	NA	5. NA	NA	NA	NA	x	NA	NA	NA	x	NA	x
	6. CCL17 (TARC) [89]	↑ (1/1)[89]	NA	NA	NA	NA	NA	NA	NA	NA	NA	6. NA	NA	NA	NA	NA	NA	NA	NA	NA	NA	NA
	7. CCL22 (MDC) [89, 139, 140]	↑ (1/1)[89]	NA	NA	NA	NA	NA	NA	NA	NA	NA	7. NA	NA	NA	NA	NA	NA	NA	NA	NA	NA	NA
	8. CXCL8 (IL-8) [135, 136, 138–142, 144–147, 155, 164, 169]	x	↑ (1/3)[145]	NA	x	↑ (3/5)[144, 145]	↑ (2/3)[145]	↑ (2/2)[144, 145]	↑ (2/4)[144, 145]	NA	x	8. ↑ (1/3)[145]	x	↑ (1/2)[145]	NA	x	x	x	NA	x	x	x
	9. CXCL9 (MIG) [135, 138, 141, 164]	x	x	NA	x	x	x	x	x	NA	NA	9. x	NA	NA	NA	x	↓ (1/1)[164]	x	NA	x	x	x
	10. CXCL13 (BLC) [135, 138]	x	x	NA	x	x	NA	NA	x	NA	NA	10. NA	NA	NA	NA	x	NA	NA	NA	x	x	↓ (1/1)[138]
Growth factors	1. M-CSF [135, 138, 140]	x	x	NA	x	↑ (1/1)[138]	NA	NA	x	NA	NA	1. NA	NA	NA	NA	↓ (1/1)[138]	NA	NA	NA	x	x	x
	2. GM-CSF [135, 136, 138–141, 144–147, 150, 155]	x	↑ (1/2)[145]	x	↑ (1/4)[138]	↑ (1/4)[138]	↓ (1/2)[144]	x	↓ (1/3)[144] ↓ (2/3)[138, 144]	NA	x	2. x	x	x	NA	↓ (1/1)[138]	NA	x	↑ (1/1)[150]	x	NA	x
	3. PDGF-BB [135, 136, 138, 141, 185]	x	x	NA	x	x	↑ (1/1)[185]	NA	x	NA	NA	3. NA	NA	NA	NA	x	NA	NA	NA	x	x	x

No associations were found with IL-1RA, IL-15, IL-33, TNF-β, TNF-sRI, CCL1 (I-309), CCL3 (MIP-1α), CCL9 (MIP-1γ), CCL24 (eotaxin-2), CXCL10 (IP-10), G-CSF, BDNF, PDGF-AA, and VEGF. Allergy and depression were found unrelated to examined analytes. Numbers in brackets stand for number of studies that confirmed analyzed association per total number of studies

*IL* interleukin, *sR* soluble receptor, *IFN* interferon, *TNF* tumor necrosis factor, *TGF* transforming growth factor, *MIF* macrophage migration inhibitory factor, *CCL* C-C motif ligand, *MIP* macrophage inflammatory protein, *MCP* monocyte chemoattractant protein, *RANTES - * regulated on activation, normal T-cell expressed and secreted, *TARC* thymus and activation-regulated chemokine, *MDC* macrophage-derived chemokine, *MIG* monokine induced by interferon-gamma, *IP-10* IFN-γ-inducible protein 10, *BLC-B* lymphocyte chemoattractant, *M-CSF* macrophage colony-stimulating factor, *GM-CSF* granulocyte-macrophage colony-stimulating factor, *PDGF* platelet-derived growth factor, *x* no association, *NA* not applicable (analyte not studied)

^a^IL-β was analyzed in all studies apart from Jácome et al. 2016 [135] and Napolioni et al. 2013 [138] who studied IL-1α, and Al-Ayadhi et al. 2005 who explored IL-1 [180]

## Pivotal role of immune system as a potential target for novel therapeutic methods

The first attempt to treat ASD on the basis of immunological disturbances came in the form of intravenous immunoglobulin (IVIG) administration. According to current guidelines, such treatment is not recommended due to limited scientific rationale [243]. However, several papers reported on improvement after IVIG. Plioplys [244] reported on 10 children who received 4 IVIG infusions with 6-week intervals, out of which 5 were considered to have improved. Four children had a partial response to treatment with regard to improved attention span and reduced hyperactivity. One child was reported to have an amelioration of autistic symptoms that regressed after IVIG discontinuation. Gupta et al. [245] treated 10 patients out of which 5 had marked improvement in prominent eye contact, echolalia, speech, and behavior. DelGiudice-Asch et al. [246] did not find any beneficial effects; however, they carried out their study on seven subjects without previous in-depth immune tests. Another study reported on 26 children who received IVIG and had improved ABC scores, and described that 22 regressed within 2–4 months after IVIG cessation [247]. An open-label study with oral encapsulated immunoglobulin therapy in ASD children with GI symptoms revealed that 50% of subjects had behavioral improvement measured with ABC and marked reduction of GI complaints [248]. However, a double-blind placebo-controlled trial showed no effectiveness of the abovementioned treatment [249]. IVIG exert an immunomodulatory effect and has been reported to be effective in several autoimmune and inflammatory disorders [250–252]. Although immunoglobulin administration has been shown to have an inhibitory effect on T-cell activation and down-regulate concentration of several cytokines [253, 254], it primarily acts on B-cell function and immunoglobulin repertoire [255]. On the basis of cellular mechanism studies and the results of human use, treatment with IVIG does not appear to hold strong potential as a disease-modifying strategy.

Interestingly, corticosteroids which have been used to treat other disorders in ASD patients were found to lessen autistic features. A child with ASD who developed autoimmune lymphoproliferative disorder improved greatly in language development and behavior after oral prednisolone therapy [242]. A retrospective analysis showed that children with regressive autism benefited from steroid therapy in language development and behavioral spheres [256, 257]. Two other cases of behavioral improvement after corticosteroid therapy were reported in ASD and PDD [258, 259]. Limitations of steroid therapy include well-known side effects and lack of expected significant improvement in core ASD domains. To date, there is only one registered clinical trial registered using pregnenolone in an attempt to lessen irritability, sensory impairment, and social sphere in autistic individuals [NCT02627508].

The US Food and Drug Administration has approved two atypical antipsychotic medications for treatment of irritability related to ASD [260]. Both risperidone and aripiprazole display interesting immunological properties in in vitro experiments. They were found to reduce proinflammatory cytokines, promote anti-inflammatory pathways, and inhibit microglial activation [261–265]. However, results from in vivo studies on cytokine changes during treatment of schizophrenia patients are inconsistent [266–269]. In children with ASD, levels of cytokine after 8 weeks of risperidone therapy were unchanged in one study [270], while Choi et al. [229] found significant reductions of two chemokines, MCP-1 and eotaxin, that have previously been reported as up-regulated. Multiple clinical trials with risperidone [NCT01171937, NCT00576732, NCT01333072, NCT00080145, NCT00584701, NCT00005014, NCT01624675, NCT00086645, NCT00374764, NCT00166595, NCT0014739] and aripiprazole [NCT00619190, NCT01333072, NCT00468130 NCT00208533 NCT00198107, NCT01028820, NCT01617447, NCT00332241, NCT00308074, NCT01227668, NCT01617460, NCT00337571, NCT00365859, NCT00198055, NCT00870727] were conducted. Other neuroleptics whose potential was explored in ASD include olanzapine [NCT00057408, NCT00183404], lurasidone [NCT01911442, NCT01620060, NCT01914393, NCT01731119], brexipiprazole [NCT03292848], and ziprasidone [NCT00208559]. Effects of neuroleptics on ASD-associated irritability and hyperactivity are promising, and though some studies report improvement in the social sphere or in stereotypy [271], most fail to address core ASD symptoms [272, 273]. Their influence on immune abnormalities is probably much more pronounced in in vitro than in vivo conditions, and although MCP-1 was found to be correlated with deficits in social sphere [150], improper communication [174], and impaired adaptive skills [138, 174], none of the fields were influenced with risperidone treatment. The possible adverse effects of neuroleptics, such as increased appetite, weight gain, fatigue, and tremor, have to be taken into consideration before deciding on treatment initiation [271–274].

Donepezil, an acetylcholinesterase inhibitor, was proven to reduce inflammatory cytokine response [275] and attenuate M1 microglia polarization [276, 277]. It was also found to be beneficial in the BTBR mouse model of ASD and valproic acid-induced mouse model of ASD [278, 279]. However, despite promising preclinical data, administration of acetylcholinesterase inhibitors such as donepezil, galantamine, or rivastigmine failed to alter concentrations of proinflammatory cytokines in peripheral blood of patients with Alzheimer’s disease [280]. In another study, four of eight patients with ASD improved on donepezil; however, the drug influenced only irritability and hyperactivity [281]. Donepezil failed to show efficacy in two double-blind, randomized clinical trials in children with ASD and Fragile X syndrome [282, 283]. Currently, there is one clinical trial open to evaluate acetyl-choline esterase inhibitors [NCT01098383].

Minocycline, a tetracycline antibiotic that could potentially alter inflammation and microglia activity [284], failed to exert clinical effects despite detected changes of hepatocyte growth factor and IL-8 in serum and BDNF changes in both serum and CSF [285]. However, in a double-blind placebo-controlled trial, minocycline as an adjunctive therapy to risperidone showed reduction of hyperactivity and irritability [286]. One clinical trial with minocycline aimed at measuring microglia activity by PET imagining is currently open [NCT03117530].

Several other medications with potent immunomodulatory properties evidenced by in vitro studies are presently under clinical trials. Currently, there are over 60 ongoing interventional clinical trials with different pharmacologic approaches and more than 130 studies already completed with no drug registered for ASD core symptoms [287].

The most currently available data still fails to reveal the most efficient mechanism of action for addressing immune abnormalities found in ASD. Regulation of cytokine expression seems a natural candidate due to vast preclinical evidence of cytokine correlation with autistic traits. Cytokine administration was also found to induce behavioral abnormalities [288, 289]. INF-α, which is known to elicit proinflammatory mediators such as IL-1, IL-2, IL-6, IL-8, and MCP-1 [290, 291], has been used for treatment of cancer and chronic viral hepatitis [292, 293] with behavioral adverse effects such as depression, anxiety, mania, and psychosis [294]. Cytokines as a therapeutic agent should be used with the utmost caution, as in vitro studies are unable to predict immune responses in living organisms due to their complicated, pleiotropic actions. Unnaturally excessive immune activation can lead to cytokine-release syndrome, a potentially life-threatening adverse effect [295].

Interestingly, a recent study in a valproic acid-exposed rat model of ASD revealed that fingolimod, an immunomodulatory agent used in clinical trials in relapsing–remitting multiple sclerosis, improved learning disturbances, memory deficits, and social impairments. It was found to reduce microglial activation and down-regulate IL-1β and IL-6 in the hippocampus [296].

Another approach is to explore the potential for utilizing stem cells in inducing immunomodulation. Recent robust research in the stem cell field revealed that stem cells have both immunomodulatory and neuroprotective potential [297]. The first clues to such association come from hematopoietic stem cell transplantation (HSCT). Interestingly, behavioral abnormalities are not transferred by bone marrow transplantation (BM-HSCT); however, they can be corrected by BM-HSCT from healthy individuals. Thus, restoring an immunophenotype could alter a disease course [12]. Stem cell intervention may be a way to correct immune system abnormalities and alternate core symptom domains of ASD.

Specific properties of mesenchymal stromal cells (MSC) make them an attractive cell source for regenerative therapy. MSCs are multipotent non-hematopoietic stem cells that display immunomodulatory properties [298]. They have been defined by the International Society for Cellular Therapy [299] as a plastic-adherent cell population with particular phenotype (CD105^+^, CD73^+^, CD90^+^, CD45, CD34^−^, CD14, CD11b^−^, CD79a^−^, CD19, HLA^−^DR^−^), able to differentiate into osteoblasts, adipocytes, and chondroblasts. They can be transplanted across allogeneic barriers because of their low immunogenicity, as they do not express major histocompatibility complex class II antigens or co-stimulatory molecules [300].

Evidence for MSC effectiveness in a mouse model has accumulated. An animal model of maternal immune activation yields offspring with autistic traits that present with increased M1 polarization, up-regulation of inflammatory cytokines and CD4^+^ response, as well as systemic Treg deficit [10–12]. The BTBR mouse strain is considered an adequate animal model for ASD due to behavioral deficits in ASD-related spheres [301]. Moreover, similar immune system abnormalities and an inflammatory phenotype are found in BTBR mice [302–304]. Intraventricular MSC transplantation in young BTBR mice ameliorated stereotypic behaviors and improved deficits in social and cognitive spheres; it is worth noting that it did alter core ASD-related symptoms. Histological analysis revealed increased neurogenesis in the hippocampal area and an elevated level of BDNF were noted. Six weeks after transplantation, MSC cells were detectable in the dorsal third ventricle [305]. Intriguingly, MSC cultured to express higher amount of BDNF were found to induce long-term effects on behavioral traits, superior to unmodified cells [306]. Intracerebral MSC administration protected mice from a social deficit induced by phencyclidine [307, 308], while adipose-derived stem cells alleviated behavioral abnormalities in a valproic acid-induced ASD model [309]. As previously mentioned, microglia in ASD individuals tend to be overly activated. Several investigators found MSC were able to inhibit microglial activation and induce neuroprotective M2 polarization [310–312]. MSC and microglial crosstalk was also investigated in vitro. MSC were found to act through expression of fractalkine (CX3CL1), which induces a neuroprotective microglia phenotype [313] and immunomodulates microglia through paracrine effects [314]. MSC are also known to express multiple neural genes and transcription factors and differentiate into neural cells after culture in a suitable media [315]. The overall safety profile from clinical applications is promising; however, it lacks long-term data [316].

A few clinical papers on stem cells use in autism have been published so far; for a summary, please see Supplementary Table 1. Firstly, a 14-year-old autistic boy [317] treated with autologous bone marrow-derived mononuclear stem cells (BM-MNC) was described. The patient had been diagnosed with severe autism with co-existing self-injurious behavior. Brain MRI was normal, PET CT scan showed reduced metabolic activity in several regions, while on EEG bilateral episodic sharp and slow wave abnormalities were seen. The patient received 56 × 10^6^ MNC intrathecally along with intensified rehabilitation. At 6 months, he was evaluated with CARS and PET CT. CARS showed substantial improvement as the boy scored 23.5 points (19-point change from the baseline), which is considered as “non-autistic.” PET CT showed increased uptake in several regions which was considered to be improvement in comparison with previous result. At 12 months, the investigators reported on further improvement, especially concerning social sphere; however, the patient was not tested with tools dedicated to evaluate autistic children. The subsequent study by Sharma et al. [318] was an open-label, proof-of-concept study with 32 patients and similar study plan; however, the patients were evaluated with Indian Scale for Assessment of Autism, Clinical Global Impression scale (CGI), and scales designed to measure independence in daily living. In CGI-II scores, all patients but one were considered as improved, including 11 who were evaluated as “very much improved.” The improvements were noted on all evaluated domains and were most pronounced in social sphere. The adverse effects related to the procedure included an increase in hyperactivity (transient in six patients, lasting over 6 months in one patient) and generalized tonic–clonic seizures in three patients that could be controlled with medications. Another study [319] used cord blood MNC along with Wharton’s jelly-derived MSC (WJ-MSC) in one of three study arms. The study employed both an intravenous and intrathecal administration route. No serious adverse effects were observed. ASD children treated with WJ-MSC yielded better results on CARS, ABC, and CGI at 24 weeks after treatment. The results were compared with patients who received cord blood-derived MNC and rehabilitation only. Unfortunately, there were no patients who received WJ-MSC without MNC. An open-labeled study for children with ASD by Bradstreet et al. [320] used a controversial stem cell source—hematopoietic stem cells derived from fetal liver (delivered intravenously) and neuroprogenitors from fetal brain tissue (delivered subcutaneously). No serious adverse effects were noted. An improvement was noted on ABC and Autism Treatment Evaluation Checklist, especially concerning speech, social, and sensory domains. Lymphocyte subpopulations were assessed at baseline, 6, and 12 months after treatment. A significant up-regulation of CD3^+^ and CD4^+^ T cells with reduced B-cell count was observed. Recently, a case series of three patients treated with human embryonic stem cells was published [321]. The patients received cells intramuscularly (once/day with a dose of approximately 4 × 10^6^), intravenously (twice/week with a dose of approximately 16 × 10^6^), and via other routes such as intrathecal administration (weekly, different dosages). Treatment was planned in 4 cycles within 4–8-month intervals. The patients were a 3-year-old boy with ASD and no other co-morbidities, a 4-year-old boy with co-existing developmental delay, and a 10-year-old boy who was also diagnosed with pediatric acute-onset neuropsychiatric syndrome, Lyme disease, heavy metal toxicity, and obsessive-compulsive disorder. The investigator concluded that patients improved significantly in eye contact, communication, cognitive skills, and writing. No information about psychological tools used to assess children were given. PET-CT examination revealed significant improvement in brain blood perfusion in all treated patients. All those results should be interpreted with caution as improvements in communication or cognitive skills are typical for young children and should be expected also in ASD individuals. For a summary of currently ongoing or unpublished clinical trials, please refer to Supplementary Table 2.

Cord blood (CB) is a unique biological material known to contain several populations of cells including progenitor stem cells, MSC, endothelial precursor cells, and unrestricted somatic stem cells [322, 323]. CB was also found to contain neurotropic and immunomodulatory factors along with several anti-inflammatory cytokines [324, 325]. The first clinical use of CB was carried out in 1988 in a setting of allogeneic HSCT in a patient with Fanconi anemia [326]. Recent advances and in-depth studies of CB biology have shown that this material can be employed in brain injuries [327]. Autologous cord blood infusion has already been shown to be safe and promising in cerebral palsy and acquired brain disorders [328, 329]. Preclinical scientific rationale support CB immunomodulatory properties and potential to correct neuronal activity [330, 331]. A breakthrough clinical trial has shown promising potential for CB and hematopoietic stem cells in ASD [332]. A phase I, open-label trial included 25 children aged 2–6 years with a confirmed ASD diagnosis and banked autologous CB. The procedure turned out to be safe and well tolerated. Improvements were noted in communication skills, expressive vocabulary, eye-tracking measures, and overall assessment of ASD severity. Interestingly, greater improvements were seen in children with higher nonverbal IQ. Further studies are planned to explore this therapeutic method, including the use of allogeneic CB.

## Future directions

**Fig. 1:**
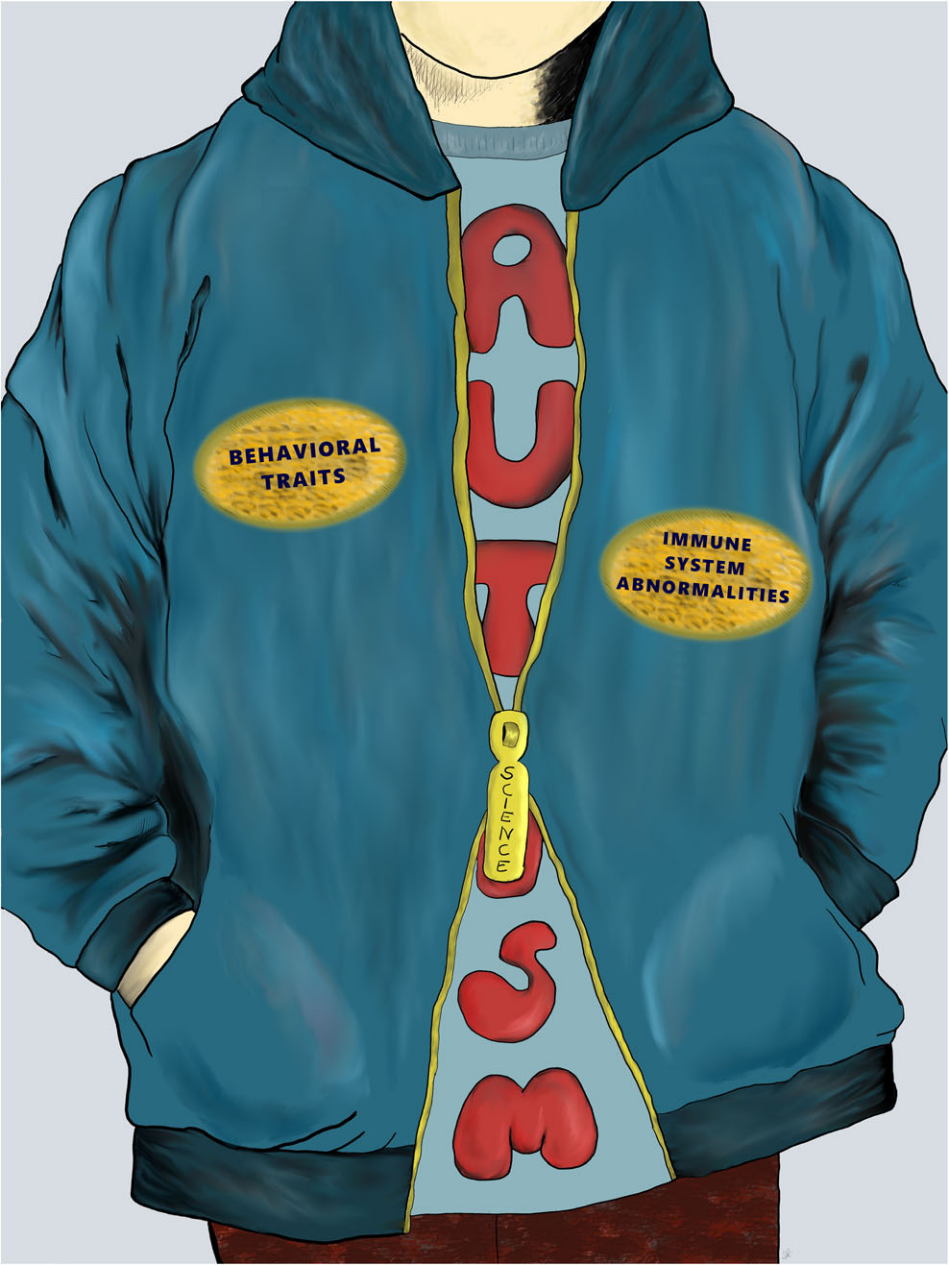
A graphic “vision” of this article

The overall data suggests that there is substantial evidence for immune system dysregulation in at least some children with ASD. The challenge lies in defining the exact connection between ASD symptoms and the immunological background. The graphic idea of this association is presented presented on Figure 1.

Studies based on newborn screening are interesting as they refer to early markers of ASD. However, they might also be misleading. Discrepancies between results of various studies may be due to methodological differences and heterogeneity of studied populations. Six studies were based on dried bloodspots from neonatal screening [65–68, 70], two on amniotic fluid [69, 72], and two on mid-gestational serum [64, 71] (Table 1).

An additional problem arises from ASD over diagnosis. In many cases, achieving a diagnosis of ASD is desirable as it facilitates parents’ ability to gain early support for children with developmental disorders [333]. Also, different periods of time (1991–2017) and advancements in ASD diagnostic ability and criteria make it difficult to compare results from early and current studies. The variety of psychological tools is of note as well, as an ASD diagnosis was not always confirmed with Autism Diagnostic Observation Schedule (ADOS) and Autism Diagnostic Interview–Revised (ADI-R), and several studies did not mention screening the control group for developmental disorders.

Out of 57 investigations that focused on cytokine and chemokine abnormalities, a detailed diagnosis of ASD was given only by 11 authors [140–142, 152, 153, 157, 159, 163, 179, 180, 183], thus clinical picture of studied subjects might have varied greatly. Patients included in the analysis could have been diagnosed with autistic disorder, Asperger syndrome, or PDD-NOS (Table 2).

Moreover, the age of subjects often greatly varied; some studies included both pediatric and adult populations, which could alter results in a significant manner. A majority of the studies were carried out on young children. Some included only preschool age [88, 94, 96, 135, 144, 145, 147, 150, 155, 158, 175, 177, 178], while others expanded inclusion criteria up to about 11 years [73, 89, 93, 142, 148, 157, 162, 163, 165, 168, 170, 172, 181]. Several studies focused solely on older children aged about 7–13 years [138, 139, 141, 146, 153, 160, 164, 166]. However, some investigators included wide-ranging age groups with not only children but also teenagers [91, 154, 156, 159, 167, 169, 171, 180, 183] and adults [136, 151]. Only three studies were carried out solely on teenagers or adults [140, 176, 182].

While mainly all control subjects were age matched, only 33 studies compared gender-matched groups, which is highly important as biomarker profiles have been found to be gender specific [140].

Another source of bias could be different methodological approaches, as studies on peripheral blood used both plasma and serum, while investigations with PB-MNC were carried out with different stimulation conditions.

Multiple drugs are known to alter the cytokine profile, but 24 groups of investigators stated that patients either did not take any medication or were taking drugs that would not compromise study results [89, 91, 94, 138, 141, 144, 145, 147, 150, 155, 160, 162, 164–166, 169–171, 174, 176, 181, 182, 185]. Several have stated that study subjects were taking psychotropic, antiepileptic, or other drugs that might be relevant [148, 151–153, 159, 168, 183]; however, almost half did not include any information on medication, which greatly impairs result interpretation.

Furthermore, ASD frequently occurs together with epilepsy, intellectual disability, ADHD, anxiety and behavior disorder, and in the course of several other diseases. The difference in cytokine profiles could be substantial as described by Jácome et al. [135] who compared ASD children with and without epilepsy. The exact data about ASD co-morbidities was frequently overlooked by investigators. Out of 57 summarized research studies on cytokine and chemokine abnormalities, 30 studies did not include any data on epilepsy, 47 on intellectual disability, and 49 on ADHD. For details, please see Table 2.

Further in-depth studies into ASD immunology could find that different behavioral traits are etiologically distinct and thus different approaches and therapeutic interventions should be undertaken. One third of conducted studies did not attempt to correlate biochemical abnormalities with behavioral traits. Observed concentrations of cytokines, chemokines, and growth factors were most frequently associated with ASD severity, impaired social interactions, and repetitive behaviors and interests. Only 3 studies, out of 26 that included psychological data, failed to detect any correlations (Table 3).

Out of multiple examined proteins, IL-1 and IL-6 turned out to be particularly interesting due to repeatability of the results concerning associated behavioral abnormalities. Probably, it could be partially attributed to the number of conducted studies in comparison to other examined molecules. IL-1 was explored by 14 groups of investigators [135, 137, 138, 144, 145, 150–152, 155, 163, 165, 166, 168, 176, 180], and IL-6 by 16 of 26 [135, 138, 144, 145, 150–152, 155, 163, 166, 167, 170–172, 176, 180]. IL-1, a key cytokine in the regulation of inflammatory pathway, was found relevant in several behavioral domains, including core ASD symptoms. It was found to be positively correlated with impaired social sphere in two [150, 155] of five studies [138, 150, 155, 166, 177]. All studies evaluated social interactions based on ADOS and ADI-R apart from Napolioni et al. [138] who employed SRS. It is worth noticing that one study analyzed cytokines on mRNA level [166], while all the others examined protein concentration with enzyme-linked immunosorbent assays. It has not escaped our notice that Careaga et al. study [150] and Enstrom et al. study [155] were conducted on children with mean age of 3, while those who did not find any correlation to social sphere were carried out on older children [138, 166] or adults [138]. Surprisingly, no overwhelming evidence over IL-1 association with severity has been found. However, out of 24 studies that explored IL-1 links with behavioral abnormalities, only 4 tried to link it with ASD severity [135, 152, 163, 180] and 1 succeeded [152]. A related point to consider is poor methodological quality of that study: wide age range (2–21 years), inclusion of children with PDD (6/29 patients), and no exact data on medication taken by subjects. On the other hand, other studies had either small [135] or heterogeneous study groups [163, 181], along with children with Rett syndrome [180]. Regression turned out to be associated in two [138, 145] of four [138, 144, 145, 163] conducted studies in this area. The topic is worth exploring since one of the studies that denied abovementioned connection was carried out after stimulation [144] and the other one included children with PDD-NOS [163]. IL-6, a complex cytokine involved in inflammation and neural functions, was found to be up-regulated in relation to social impairment in half [150, 155, 167] of the conducted studies [138, 150, 155, 166, 167, 176]. Analogically to IL-1, IL-6 was found significant in younger children [150, 155] or in a large pediatric study group [167]. No correlation with cognition has been made by the same investigators who explored also IL-1 [138, 166, 177]. Intriguingly, three [135, 152, 171] of four [135, 152, 163, 171] studies reported up-regulation of IL-6 in association with disease severity. Single studies have linked up-regulation of IL-1 and IL-6 with repetitive behaviors and interests [145], impaired communication [155], development [150], adaptive skills [138], aggression [150], hyperactivity and irritability [151], or lower IQ [138]. Despite vast studies of both IL-1 and IL-6, no associations with cognitive sphere have been reported so far. It is worth underlying that up-regulation of IL-1 or IL-6 and their connection to social sphere was significant in young individuals [150, 155]. It would be beneficial to examine large groups of children before psychological interventions, just after establishing diagnosis. We could suspect that those children would manifest the most prominent behavioral abnormalities and thus become an aim of further in-depth studies.

The search for potential biomarkers and their correlation with phenotypic variability should be the point of focus in ASD research and make a ground for future targeted therapies. Singh [334] hypothesized that an autoimmune autistic disorder might be identified and treated accordingly. However, his hypothesis involved mainly virus-induced autoimmunity. Several other interesting factors such as leptin, osteopontin, cell adhesion molecules, markers of oxidative stress, and neurotransmitters were reported to be relevant in ASD [335–340].

Not every study was in favor of an ASD immune pathogenesis. Stern et al. [117] concluded that only 2 out of 24 examined individuals had altered immune function, out of which 1 had common variable immune deficiency and routine immunologic examination would not benefit this group of patients. However, it is worth noticing that Stern et al. [117] also included children with PDD and the age of included patients varied from 3 to 17 years. For a full list of excluded correlations and a summary of studies conducted on peripheral blood, please refer to Table 3.

Taken together, the presented data suggest a strong link between autism and immune dysfunction. Caution in drawing a conclusion should be preserved due to the lack of consistency in the studied populations, as the variety of co-existing symptoms and neurological comorbidities makes it difficult to completely synthesize all conducted studies. The association between immune system dysfunction and behavioral abnormalities, in at least a subset of individuals with ASD, suggests a potential role for immunomodulatory therapies as a causative treatment. Several investigators have already reported on the first clinical uses of stem cells in patients with ASD with promising results [317–319, 332]. Cellular therapies that take advantage of immunomodulatory properties of stem cells could address neurodevelopmental abnormalities on a cellular level. A summary of ongoing or unpublished clinical trials is presented in Supplementary Table 2.

It is noteworthy that in the future, we may be able to redefine ASD on the basis of molecular, immunological, and biochemical background and determine patients who could benefit from immunomodulatory approach.

## Electronic supplementary material

Supplementary Table 1 (DOCX 17 kb)

Supplementary Table 2 (DOCX 17 kb)
