# Supplementary material for: Immune Abnormalities in Autism Spectrum Disorder—Could They Hold Promise for Causative Treatment?
Source: Mol Neurobiol. 2018 Jan 6;55(8):6387–435. doi: 10.1007/s12035-017-0822-x (PMC6061181; doi:10.1007/s12035-017-0822-x)
Supplement: Supplementary file 1 — (DOCX 17 kb) [file 12035_2017_822_MOESM1_ESM.docx]

Supplementary Table 1. Clinical cases of stem cell application in autism spectrum disorders.

| Study details | Study | Sharma et al. 2013 [317] | Sharma et al. 2013 [318] | Lv et al. 2013 [319] | Bradstreet et al. 2014 [320] | Shroff 2017 [321] |
| --- | --- | --- | --- | --- | --- | --- |
|  | Country | India | India | China | USA, Ukraine, Italy | India |
|  | Type and design | case report | open-label proof of concept study | clinical trial I/II, open-label, non-randomized | open-label pilot study | case series |
|  | Control group | no | no | 1) only rehabilitation (n=14) 2) rehabilitation+MNC (n=14) | no | no |
| Patients' history | Subject number | 1 | 32 | 9 (receiving both MNC and MSC) | 45 | 3 |
|  | Gender | male | 24 males, 8 females | males | 39 males, 6 females | 3 males |
|  | Age at therapy [years] | 14 | 3-33, mean: 10.49 | 4-10; mean: 6.2 | 3-15, mean: 6.94 | 3-, 4- and 10-years-old |
|  | Co-existing epilepsy | no | yes, number of patients not specified | no data | no | no |
|  | Regression | no | no data | no data | no data | no exact data |
|  | Milestones | deleyed speech | no data | no data | no data | deleyed |
| Examinations | Neurological examination | no abnormalities | no data | no data | no abnormalities | no data |
|  | EEG study | bilateral episodic sharp and slow wave abnormalities | no exact data | x | x | x |
|  | MRI | no signicifant abnormalities | no data | x | x | x |
|  | Brain PET | reduced metabolic activity in several regions | no base-line data | x | x | x |
|  | CARS | 42.5 (severe) | x | 45.11 ± 4.31 | no data | x |
|  | ISAA | x | median of 115.5 | x | x | x |
|  | ABC | x | x | 91.78 ± 25.92 | 80 ± 2 | x |
|  | ATEC | x | x | x | 83 ± 5.5 | x |
|  | Immunological studies | x | x | immunoglobulins, T cell subsets | lymphocyte subpopulations | x |
| Stem cell therapy | Stem cell donor | autologous | autologous | allogeneic | allogeneic | allogeneic |
|  | Source of stem cells | bone marrow afer G-CSF mobilization | bone marrow afer G-CSF mobilization | cord blood and umbilical cord | human fetuses | human ebryons |
|  | Type of cells | MNC | MNC | MNC + MSC | HSC derived from fetal liver, neuroprogenitor cells derived from fetal brain | ESC |
|  | Delivery route | intrathecal | intrathecal | intrathecal, intravenous | intravenous , subcutaneus | intravenous, intramuscular, intrathecal and others |
|  | Number of stem cells | 5.6x10^6 | 8.19 × 10^7 (mean) | 2×10^6/kg MNC + 1×10^6/kg MSC | i.v.:1.6ml of suspension with >30 × 10^6 cells/ml; s.c.: 2.12±0.49ml with >8.70×10^6 cells/ml | depended on administration route |
|  | Number of interventions | 1 | 1 | 4xMNC, 2xMSC | 2 | 4 phases |
|  | Interval between doses | x | x | 5-7 days | 1 day | 4-6 months |
|  | Side effects | none | several procedure-related and three related to the stem cell therapy: seizures and transient or persistent increase in hyperactivity | low-grade fever that subsided spontanously | none | no data |
| Follow-up | Follow-up period | 12 months | 5 to 26 months (mean: 12.7) | 24 weeks | 12 months | no exact data |
|  | Follow-up examinations | CARS, PET at 6 months | ISAA, CGI, FIM/Wee-FIM, PET-CT | CARS, ABC, CGI | ABC, ATEC | PET |
|  | Results | CARS score dropped to 23.5 from 42.5; PET CT: increased uptake in: temporal lobes, calcarine cortices and left medial pre-frontal cortex | Patients improved on CGI-I and ISAA (all domains), no improvement concerning FIM/Wee-FIM. PET-CT in 8 patients showed normalization of FDG uptake in several regions | Patients improved on CARS (from 45.11±4.31 to 28.00±6.18), ABC (from 91.78 ± 25.92 to 36.78±16.95) and CGI, no difference in immunological studies was noted | Patients improved on ATEC (from 83 ± 5.5 to 59±9.5) and CARS (from 80±2 to 54±2.9), immunological studies revealed decreased B-lymphocyte and increased T-helper percentage | Improvement in several domains: eye coordination, writing, cognition, speech |

Abbreviations: G-CSF - granulocyte colony-stimulating factor, CARS - Childhood Autism Rating Scale, PET - Positron Emission Tomography, CT - Computed Tomography, ISAA - The Indian Scale for Assessment of Autism, CGI – Clinical Global Impression Scale, FIM - Functional Independence Measure, FDG - fluorodeoxyglucose, MNC – mononuclear cells, MSC – mesenchymal stromal cells, ABC – Aberrant Behavior Checklist, HSC – hematopoietic stem cells , ATEC - Autism Treatment Evaluation Scale , ESC – embryonic stem cells
