# Supplementary material for: Immune Abnormalities in Autism Spectrum Disorder—Could They Hold Promise for Causative Treatment?
Source: Mol Neurobiol. 2018 Jan 6;55(8):6387–435. doi: 10.1007/s12035-017-0822-x (PMC6061181; doi:10.1007/s12035-017-0822-x)
Supplement: Supplementary file 2 — (DOCX 17 kb) [file 12035_2017_822_MOESM2_ESM.docx]

Supplementary Table 2. Ongoing or unpublished clinical trials of stem cell application in autism spectrum disorders.

| Study details | Study | NCT01836562 | NCT02627131 | NCT03225651 | NCT01740869 | NCT02192749 | NCT01974973 | NCT01502488 |
| --- | --- | --- | --- | --- | --- | --- | --- | --- |
|  | Current status | unknown | completed | recruiting | unknown | ongoing | completed | withdrawn |
|  | Country | India | Vietnam | Vietnam | Mexico | Panama | India | Mexico |
|  | Type and design | clinical trial I/II, open label, non-randomized | clinical trial II, open label, non-randomized | clinical trial II, open label, non-randomized | clinical trial I/II, open label, non-randomized, with crossover assignment | clinical trial I/II, open label, non-randomized, with crossover assignment | clinical trial phase I, open label, non-randomized | clinical trial phase I/II, open label, non-randomized |
| Patients' history | Subjects | 100 | 24 | 60 | 30 | 20 | 150 with any type of ASD | 10 |
|  | Gender | no data | no data | no data | no data | no data | no data | no data |
|  | Age at enrollment [years] | 3-70 | 3-16 | 3-7 | 5-15 | 6-16 | 0.5-40 | 3-12 |
|  | Co-existing epilepsy | no data | no data | no data | no data | no | no data | no active seizures |
| Examinations | EEG study | x | X | X | X | x | x | x |
|  | MRI | x | X | X | X | x | x | x |
|  | Brain PET | yes | X | X | X | x | yes | x |
|  | Psychological tools | CARS | CARS | CARS, ISAA, ADOS | CARS | CARS, ATEC | no data | CARS (≥ 30 points at baseline), ABC, CGI |
|  | Immunological studies | x | X | X | X | TARC and MDC concentration in several timepoints | x | x |
| Stem cell therapy | Stem cell donor | autologous | autologous | autologous | autologous | allogeneic | autologous | autologous |
|  | Source of stem cells | bone marrow | bone marrow | bone marrow | bone marrow after G-CSF mobilization | cord tissue | bone marrow | adipose tissue |
|  | Type of cells | MNC | MNC | MNC | MNC | MSC | MNC | ASC |
|  | Delivery route | intrathecal | Intrathecal | Intrathecal | intrathecal | intravenous | intravenous | intravenous |
|  | Number of stem cells | 100x10^6 | no data | no data | no data | no data | no data | no data |
|  | Number of interventions | 3 | 2 | 2 | 1 | 4 | 1 | 1 |
|  | Interval between doses | 10 days | 3 months | 6 months | x | 3 months | x | x |
| Results | Follow-up period | 6 months | 6 months | 12 months | 6 months | 6 months | 6 months | 6 months |
|  | Follow-up examinations | CARS, PET | CARS | CARS, ADOS, ISAA | CARS, PET | CARS, ATEC | PET, psychologic tools at 6 months | CARS, ABC, CGI at 3 and 6 months |

Abbreviations: EEG - electroencephalography, MRI - magnetic resonance imaging, PET - Positron Emission Tomography, , CARS - Childhood Autism Rating Scale, MNC – mononuclear cells, ISAA - The Indian Scale for Assessment of Autism, ADOS - Autism Diagnostic Observation Schedule, ATEC - Autism Treatment Evaluation Scale, G-CSF - granulocyte colony-stimulating factor, MDC - macrophage-derived chemokine, TARC - thymus- and activation-regulated chemokine, MSC - mesenchymal stromal cells, ASD - autism spectrum disorders, ABC – Aberrant Behavior Checklist, CGI – Clinical Global Impression Scale, ASC – adipose-derived stem cells.
